# Enhancing the biosynthesis of taxadien-5α-yl-acetate in *Escherichia coli* by combinatorial metabolic engineering approaches

**DOI:** 10.1186/s40643-024-00762-8

**Published:** 2024-05-16

**Authors:** Wen-Liang Xie, Mei-Fang Zhang, Zheng-Yu Huang, Man Xu, Chun-Xiu Li, Jian-He Xu

**Affiliations:** Laboratory of Biocatalysis and Synthetic Biotechnology, State Key Laboratory of Bioreactor Engineering, East China University of Science and Technology, 130 Meilong Road, Shanghai, 200237 People’s Republic of China

**Keywords:** *Escherichia coli*, Methylerythritol phosphate pathway, Taxadien-5α-yl-acetate, Taxadien-5α-ol *O*-acetyltransferase, Multivariate-modular metabolic engineering

## Abstract

Biosynthesis of paclitaxel (Taxol™) is a hot topic with extensive and durable interests for decades. However, it is severely hindered due to the very low titers of intermediates. In this study, *Escherichia coli* was employed to *de novo* synthesize a key intermediate of paclitaxel, taxadien-5α-yl-acetate (T5OAc). Plasmid-based pathway reconstruction and optimization were conducted for T5OAc production. The endogenous methylerythritol phosphate pathway was enhanced to increase the precursor supply. Three taxadien-5α-ol *O*-acetyltransferases were tested to obtain the best enzyme for the acetylation step. Metabolic burden was relieved to restore cell growth and promote production through optimizing the plasmid production system. In order to achieve metabolic balance, the biosynthesis pathway was regulated precisely by multivariate-modular metabolic engineering. Finally, in a 5-L bioreactor, the T5OAc titer was enhanced to reach 10.9 mg/L. This represents an approximately 272-fold increase in production compared to the original strain, marking the highest yield of T5OAc ever documented in *E. coli*, which is believed to be helpful for promoting the progress of paclitaxel biosynthesis.

**Supplementary Information:**

The online version contains supplementary material available at 10.1186/s40643-024-00762-8.

## Keypoints

We used *E. coli* BL21 (DE3) to *de novo* synthesize T5OAc.

Increasing precursor and relieving metabolic stress to enhance T5OAc production.

The modular metabolic engineering strategy achieved the balance of metabolic flux.

**Supplementary Information:**

The online version contains supplementary material available at 10.1186/s40643-024-00762-8.

## Introduction

The diterpenoid alkaloid, paclitaxel, is renowned for its potent anticancer properties and has been extensively utilized in the therapeutic management of a broad spectrum of malignancies (Miele et al. 2012; Harry and Long 1994). However, only 1 g of pure product can be obtained from three mature *Taxus* trees, which is far from meeting the clinical needs (Wheeler et al. 1992; Nadeem et al. 2002). In view of this issue, chemists began to study the chemical synthesis of paclitaxel. Due to the complex synthesis route, difficult reaction conditions and extremely low synthesis rate, the total synthesis could not be industrialized (Nicolaou et al. 1994; Nikolic et al. 2011). The semi-synthetic method, involving the simple chemical modification of the paclitaxel intermediates to synthesize paclitaxel, became the primary approach for the industrial production of paclitaxel. However, this method still relies on yew plants (Shao et al. 2021).

**Fig. 1:**
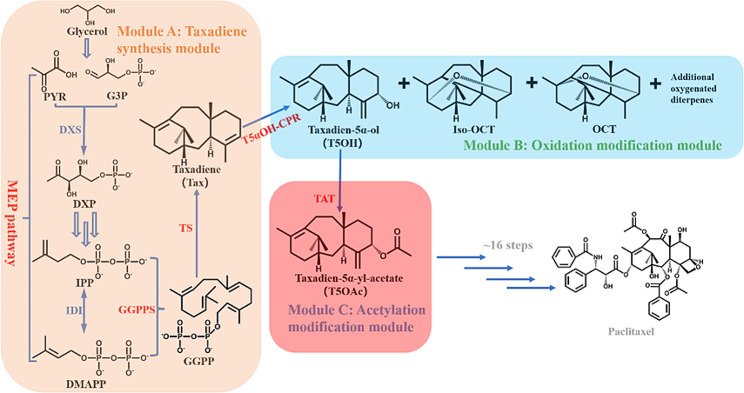
Modular engineering for the production of T5OAc in *E. coli*. The three modules are designated for the synthesis, oxidation, and acetylation of Tax, respectively. Endogenous pathway enzymes and heterogenous pathway enzymes are shown in blue and red, respectively. MEP: methylerythritol phosphate; PYR: pyruvate; G3P: glyceraldehyde 3-phosphate; DXP: 1-deoxy-D-xylulose 5-phosphate; IPP: isopentenyl diphosphate; DMAPP: dimethylallyl diphosphate; GGPP: geranylgeranyl diphosphate; Iso-OCT: 5(13)-oxa-3(11)-cyclotaxane; OCT: 5(12)-oxa-3(11)-cyclotaxane; DXS: 1-deoxy-D-xylulose-5-phosphate synthase; IDI: isopentenyl diphosphate isomerase; GGPPS: geranylgeranyl diphosphate synthase; TS: taxadiene synthase; T5αOH-CPR: taxadien-5α-hydroxylase truncated 24 amino acids is fused with its truncated transmembrane region reductase chaperone; TAT: taxadien-5α-ol *O*-acetyltransferase

The heterologous biosynthesis of paclitaxel has enormous advantage in solving resource scarcity, which has attracted wide attention (Wang et al. 2022; Ding et al. 2014; DeJong et al. 2006; Li et al. 2019). Starting from taxadiene (Tax), at least 18 reactions are required for biosynthesis of paclitaxel, including multiple enzyme modification steps mediated by cytochrome P450s and acyltransferases (Li et al. 2019) (Fig. 1). Tax, is derived from the isopentenyl diphosphate (IPP) and its isomer, dimethylallyl diphosphate (DMAPP). IPP and DMAPP can be synthesized through the methylerythritol phosphate (MEP) pathway or mevalonate (MVA) pathway. Isoprenyl diphosphate isomerase (IDI) enables the reversible interconversion of IPP and DMAPP (Hampel et al. 2005; Daletos et al. 2020). IPP and DMAPP are enzymatically converted into geranylgeranyl diphosphate (GGPP) by the action of geranylgeranyl diphosphate synthase (GGPPS), which then form Tax under the catalysis of taxadiene synthase (TS) (Huang et al. 2021). Tax is tailored by taxadien-5α-hydroxylase (CYP725A4, T5αOH) to form taxadien-5α-ol (T5OH) and other oxygenated products (By-products), such as 5(12)-oxa-3(11)-cyclotaxane (OCT), 5(13)-oxa-3(11)-cyclotaxane (*iso*-OCT). Subsequently, T5OH is acetylated by taxadien-5α-ol *O*-acetyltransferase (TAT) to form taxa-4(20),11-dien-5α-yl-acetate (T5OAc) (Walker et al. 1999). Tax, along with its various chemically modified derivatives, is collectively classified under the group known as taxanes. Finally, through multiple steps of hydroxylation, acetylation and side chain modification, T5OAc is transformed to paclitaxel (Fig. 1).

Metabolic pathway flux and key enzyme optimization strategies were applied for biosynthesis of Tax and oxygenated taxanes in *E. coli* MG1655 (DE3), and the titers recorded were 1 and 0.57 g/L, respectively (Ajikumar et al. 2010; Biggs et al. 2016). The titers of Tax and oxygenated taxanes were 129 mg/L and 199 mg/L, as achieved by enhancing the TS solubility and optimizing the fermentation condition in *Saccharomyces cerevisiae* (Nowrouzi et al. 2020; Walls et al. 2022). However, the next acylation step to form T5OAc hindered the progress of paclitaxel biosynthesis. Currently, the highest titers of T5OAc have been obtained using the *S. cerevisiae* LR6 strain. This strain achieved T5OAc levels of 26 mg/L through *in silico* design and 95 mg/L using a ten-day semi-continuous cultivation combined with combinatorial resin adsorption (Malci et al. 2023; Santoyo-Garcia et al. 2023). It was noteworthy that the LR6 strain was a genetically engineered variant, specifically designed for T5OAc production via extensive metabolic engineering (Walls et al. 2020). In bioreactor condition, the strain’s T5OAc production was limited to 3.7 mg/L and was undetectable in shake-flask culture. This underscored the necessity for multiple rounds of metabolic engineering and optimization to develop highly efficient T5OAc-producing strain. In contrast, *E. coli* was reported to produce up to 1 g/L of Tax, highlighting its potential as a host for synthesizing paclitaxel intermediates (Ajikumar et al. 2010). However, research on T5OAc production in *E. coli* remained scarce, only less than 1 mg/L of T5OAc was detected in *E. coli* MG1655 (DE3) (Edgar et al. 2016). The primary reason for achieving such low titer was attributed to the utilization of a chassis that was not the optimized engineered strain, referred to as strain 7, known for its high production of oxygenated taxanes (Biggs et al. 2016). Additionally, another factor was the absence of fermentation conditions optimization. Based on prior research, *E. coli* MG1655 (DE3) exhibited the highest titers of Tax and oxygenated taxanes (Ajikumar et al. 2010; Biggs et al. 2016), indicating that *E. coli* was suitable as a powerful host for T5OAc production. Furthermore, the ratio of T5OH to the total oxygenated taxanes was 26% in *E. coli* BL21 (DE3) through regulation, a value surpassing the 7% observed in MG1655 (DE3). (Wu et al. 2022; Edgar et al. 2016). In view of the above facts, *E. coli* BL21 (DE3) was strategically selected as the host organism for the biosynthesis of T5OAc.

In this study, *E. coli* was applied to *de novo* synthesis of T5OAc from glycerol, and further increased its titer by enhancing the supply of precursors, screening key enzyme, and relieving the metabolic burden. Finally, we achieved an efficient engineered strain with a balance of metabolic pathway flux through multivariate-modular metabolic engineering and culture condition adjustment. The titer of T5OAc reached 10.9 mg/L in bioreactor, this is the highest reported among engineered *E. coli* strains. The cell factory constructed here is expected to be helpful for promoting the development of paclitaxel biosynthesis.

## Materials and methods

### Strains and genes

In our study, *E. coli* TOP10 and BL21 (DE3) (Novagen, Germany) were utilized for the purposes of cloning and expression, respectively. Detailed information regarding the strains employed and those developed as part of this research were comprehensively outlined in Table S1. All genes and their sources employed in our research were presented in Table S2. GGPPS, TS, T5αOH and Cytochrome P450 reductase (CPR) were stored in our laboratory, *Tc*TAT1, *Tc*TAT2, and *Ca*TAT were synthesized by GenScript (Nanjing, China) after codon-optimized.

### Plasmids and DNA manipulation

Comprehensive information regarding the plasmids and primers employed in this study can be found in Tables S3 & S4, respectively. Empty vectors pET-21a (Novagen, Germany), pRSFDuet-1 (Novagen, Germany), pACYCDuet-1 (Novagen, Germany), pCWori (Zomanbio, China), pTrcHis2B (Invitrogen, Britain) and pQE-30 (Zomanbio, China) were employed to construct recombinant vectors. All plasmids were assembled and validated using the ClonExpress Ultra One Step Cloning Kit (Vazyme, China) and Sanger sequencing (Tsingke, China), respectively.

In the plasmids fortified for the MEP pathway, p40T7-IDI were constructed by amplifying the IDI gene with primers 1 F&R and the linearized vector pET-21a with primers 2 F&R. Plasmid p40T7-DXS-DXR-IDI was constructed by amplifying the DXR gene with primers 3 F&R and the linearized vector p40T7-DXS-IDI with primers 4 F&R. Plasmid p40T7-DXS-IDI-ISPD-ISPF was constructed by amplifying the ISPD/ISPF genes with primers 5–6 F&R and the linearized vector p40T7-DXS-IDI with primers 7 F&R.

For heterologous gene expression vectors, plasmid pATG was generated by amplifying the TS gene with primers 8 F&R, the GGPPS gene with primers 9 F&R, the linearized fragment between the dual T7 promoters of empty vector pACYCDuet-1 with primers 10 F&R, and the residual linearized vector pACYCDuet-1 with primers 11. Plasmid pRTC was generated by amplifying the T5αOH-CPR gene with primers 12 F&R and the linearized vector pRSFDuet-1 with primers 13 F&R. Plasmids pATCT1-3 were constructed by amplifying the *Tc*TAT1-2/*Ca*TAT genes with primers 14–16 F&R and the linearized vector pACYCDuet-1-T5αOH-CPR with primers 17–19 F&R. Plasmid pATGTCT2 was constructed by amplifying the GGPPS gene with primers 20 F&R, the linearized fragment between TS and GGPPS within the pATG vector with primers 21 F&R, the T5αOH-CPR-*Tc*TAT2 gene with primers 22 and the residual linearized vector pATG with primers 23. Plasmid pRTGTCT2 was constructed by amplifying the TS-GGPPG-T7- T5αOH-CPR-*Tc*TAT2 fragment from pATGTCT2 with primers 24 F&R and the linearized vector pRSFDuet-1 with primers 25 F&R. Plasmids pADITGTCT2/pRDITGTCT2 were constructed by amplifying the DXS-IDI fragment from p40T7-DXS-IDI with primers 26–27 F&R and the linearized vector pATGTCT2/pRTGTCT2 with primers 28–29 F&R. Plasmid pADITG was constructed by amplifying the DXS-IDI fragment from p40T7-DXS-IDI with primers 30 F&R, the TS-GGPPS fragment from pATGTCT2 with primers 31 F&R and the linearized vector pACYCDuet-1 with primers 32 F&R. Plasmid pADITGTC was constructed by amplifying the T5αOH-CPR fragment from pACYCDuet-1-T5αOH-CPR with primers 33 F&R and the linearized vector pADITG with primers 34 F&R.

For the section on promoter replacement experiments, the plasmids pRTrc/T5 were constructed by amplifying the promoters Trc/T5 from pTrcHis2B/pQE30 with primers 35–36 F&R and the linearized vector pRSFDuet-1 with primers 37–38 F&R. Plasmids p20TrcT2/ p20T5T2/ p20T7T2 were constructed by amplifying the *Tc*TAT2 gene with primers 39–41 F&R and the linearized vectors pRTrc/T5/pRSFDuet-1 with primers 42–44 F&R. Plasmids pCTacT2/ pTT7T2 were constructed by amplifying the *Tc*TAT2 gene with primers 45–46 F&R and the linearized vectors pCWori/pET-21a with primers 47–48 F&R. The construction process of double promoter replacement vectors derived from pACYCDuet-1 were rather intricate. Here, we used p10TrcDITGTrcTC as an illustrative example. Firstly, plasmids pTrcHis-DITG/pTrcHis-TC were generated by amplifying the DXS-IDI-TS-GGPPS/T5αOH-CPR fragment from pADITGTC with primers 49–50 F&R and the linearized vectors pTrcHis2B with primers 51–52 F&R. Subsequently, plasmid p10TrcDITG was generated by amplifying the Trc-DXS-IDI-TS-GGPPS fragment from pTrcHis-DITG with primers 53 F&R and the linearized vectors pACYCDuet-1 with primers 54 F&R. Finally, plasmid p10TrcDITGTrcTC was generated by amplifying the Trc-T5αOH-CPR fragment from pTrcHis-TC with primers 55 F&R and the linearized vectors p10TrcDITG with primers 56 F&R.

### Culture conditions

In order to obtain recombinant strains, we successfully introduced the constructed recombinant plasmids into *E. coli* BL21(DE3) using the heat-shock method. Single colony was selected and inoculated in lysogeny broth (LB) test-tube with an appropriate amount of antibiotics (ampicillin: 100 mg/L, kanamycin: 50 mg/L, chloramphenicol: 34 mg/L), then oscillated at 37 °C /200 rpm for 12–16 h to obtain preculture. For taxanes production in shake-flask, the preculture was inoculated at a 1% (v/v) ratio into shake-flask (250 mL) with terrific broth (TB) medium (50 mL), and 2% (w/v) glycerol and appropriate antibiotics were further added. The culture was incubated at 37 °C with agitation at 200 rpm. Upon reaching an OD_600_ of 0.8, inducers, specifically 0.1 mM isopropyl *β*-d-1-thiogalactopyranoside (IPTG) and 0.2 mM δ-aminolevulinic acid (ALA) were added to facilitate protein expression. Subsequently, the incubation temperature was lowered to 18 °C and the cultivation was continued for 72 h with/without a cover of 10% *n*-dodecane. When the fermentation conditions were optimized, specific factor was adjusted while all other conditions remained unchanged.

For fed-batch fermentation, the preculture was first inoculated into shake-flask (2 L) with LB medium (300 mL) at 37 °C/200 rpm for 4–5 h. When the OD_600_ reached 2, the culture was introduced into a 5-L bioreactor containing 3 L medium (20 g/L glycerol, 10 g/L Tryptone, 5 g/L yeast extract, 5 g/L Na_2_HPO_4_·12H_2_O, 0.7 g/L Na_2_SO_4_, 3.4 g/L KH_2_PO_4_, 0.25 g/L MgSO_4_, 2.7 g/L NH_4_Cl). Fermentation was initiated at 37 °C, and hydrochloric acid (3 M) and ammonia (50%) were employed for pH control at 7.0. Dissolved oxygen (DO) was maintained above 30% air saturation by correlating it with the stirring speed. When OD_600_ reached 15, inducers (consistent with shake-flask cultivation) were introduced into the bioreactor. Following this addition, the temperature of the bioreactor was lowered to 18 °C to initiate induction. Meanwhile, a feeding (medium: 200 g/L glycerol, 60 g/L tryptone and 30 g/L yeast extract) strategy based on the DO-Stat method was used to sustain cell growth. Specifically, when DO exceeded 40% air saturation, feeding was initiated at a rate of 3 L/h. During the fermentation process, samples were taken every 12 h for the measurement of OD_600_ and products analysis.

### Analytical procedures

The standard compound of Tax was stored in our laboratory, while T5OAc was separated and purified in this study. Tax and T5OAc were quantified using authentic standards, while the other products (T5OH and by-products) were relatively quantified based on standard Tax concentrations. In the biphasic fermentation process utilizing *n*-dodecane, the fermentation broth underwent centrifugation at a force of 13,000 *g* for a duration of 5 min. Subsequently, the *n*-dodecane phase was meticulously separated and amalgamated with an ethyl acetate, which was supplemented with *n*-octadecane at a concentration of 10 mg/L as an internal standard. This mixture was specifically prepared for analytical using gas chromatography-mass spectrometry (GC-MS). In one-phase fermentation, the products were extracted using an equal volume of ethyl acetate containing internal standards, followed by stirring for 10 min. Then, the resultant mixture was subjected to centrifugation at 13,000 *g* for a period of 5 min, after which the ethyl acetate layer was carefully extracted for subsequent GC-MS analysis. The analysis program was conducted that commenced at an initial temperature of 80 °C, maintained for 3 min. Subsequently, the temperature was ramped up at a rate of 20 °C per minute to reach 250 °C, which was then maintained for 3 min.

### Separation and identification of T5OAc

Subsequent to the completion of the fed-batch fermentation, the resulting fermentation broth was amalgamated with ethyl acetate. This mixture was then subjected to a thorough agitation process, lasting for a duration of 30 min. Then, the ethyl acetate phase was carefully extracted and then concentrated under vacuum. Silica gel column chromatography was utilized for the preliminary separation, *n*-hexane and ethyl acetate in a ratio of 120:1 as the mobile phase, and semi-preparative liquid chromatography was used for further purification to obtain pure T5OAc (8 mg). The T5OAc was dissolved using C_6_D_6_ for nuclear magnetic resonance (NMR) analysis (Supplementary Figures S1-S2). The standard curve for T5OAc has been developed, with 10 mg/L *n*-octadecane added as the internal standard, as shown in Supplementary Figure S3.

### **Estimation of enzyme expression level and SDS-PAGE**

The expression level was obtained by multiplying the copy number on the plasmid and the promoter strength (Wu et al. 2013). The copy numbers of plasmid and promoter used were assigned (Jones et al. 2020; Sorensen et al. 2005; Wu et al. 2022). The copy numbers of pACYCDuet-1 (p15A Ori) were 10 arbitrary units (a.u.), and the numbers of pRSFDuet-1 (RSF Ori) were 20 a.u. The promoters T7/T5/Trc strengths were 5/2/1, respectively (Brosius et al. 1985; Brunner et al. 1987). The SDS-PAGE analysis method refers to previous research (Zhang et al. 2023).

## Results

### **Designing the** ***de novo*** **Biosynthetic Pathway for T5OAc in** ***E. coli*** **BL21 (DE3)**

**Fig. 2:**
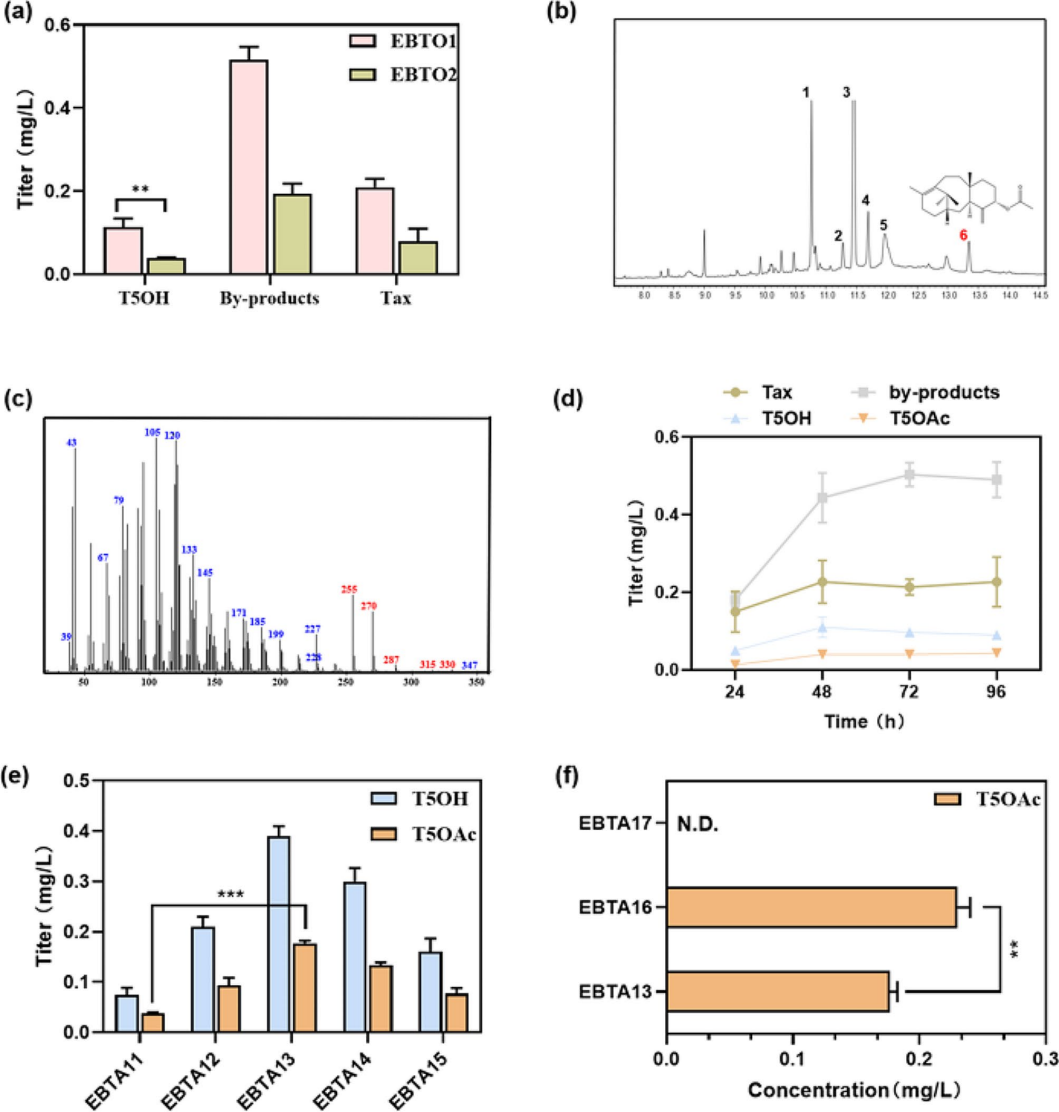
Construction and Optimization Strategies for T5OAc-producing strains. **(a)** By expressing heterologous enzymes (TS-GGPPS and T5αOH-CPR) on different plasmids (p15A or RSF Ori), two kinds of engineered strains with different synthetic ability of T5OHwere constructed. **(b)** GC-MS analysis of the EBTA11 strain revealed six distinct compounds: taxadiene (1), taxadiene-5α-ol (5), By-products (2, 3, 4) and taxadien-5α-yl-acetate (6). **(c)** Mass spectral fragmentation pattern of the biosynthetic product of the strain EBTA11 fermentation. The spectrum is identical to that of authentic taxadien-5α-yl-acetate (T5OAc); the ions at m/z 330 (P^+^), 315 (P^+^-CH_3_), 287 (P^+^-CH_3_CO), 270 (P^+^-CH_3_COOH), and 255 (P^+^-CH_3_COOH-CH_3_) are diagnostic. **(d)** Batch fermentation (250 mL shaking-flask) process of the strain EBTA11. **(e)** Based on strain EBTA11, the MEP pathway was augmented to varying extents, resulting in the generation of distinct strains EBTA12-15. **(f)** Effect of TAT from different sources on production concentration. The experiments were conducted in triplicate, and the results are presented with error bars representing standard deviations. Significance (*p*-value) was evaluated by two-sided t-test, ** presents *p* < 0.01, *** presents *p* < 0.001. N.D., not detected

In the endeavor to develop a T5OAc-producing strain, we initially developed two T5OH-producing strains to serve as precursor sources. Recombinant vectors, namely pRSFDuet-1-TS-GGPPS and pACYCDuet-1-T5αOH-CPR, were designed for the expression of TS-GGPPS and T5αOH-CPR, respectively (Wu et al. 2022). Additionally, vectors pATG and pRTC were generated to express TS-GGPPS and T5αOH-CPR separately, facilitating the assessment of the plasmid copy number’s impact on pathway enzymes. The corresponding recombinant vectors were introduced into *E. coli* BL21(DE3) to yield T5OH-producing strains EBTO1 and EBTO2 (Table S1). Small-scale (50 ml) fermentation experiments for 72 h were performed on EBTO1 and EBTO2 strains with a cover of 10% (v/v) *n*-dodecane to extract products in situ. The titers of T5OH in EBTO1 and EBTO2 reached 0.11 and 0.04 mg/L (Fig. 2a). Then, based on the EBTO1 strain, the T5OAc-producing strain, EBTA11, was generated by tandem expression of TAT via the ribosome binding site (RBS: GAAGGAGATATACAT) after T5αOH-CPR (Table S1). The GC-MS analysis showed that the T5OAc was formed in the *n*-dodecane phase (Fig. 2b and c). After 72 h of shake-flask fermentation, the highest titer of T5OAc reached 0.04 mg/L (Fig. 2d and e).

IPP and DMAPP, the pivotal precursors for terpenoid biosynthesis, are synthesized via the natural MEP pathway in *E. coli* (Martin et al. 2003). DXS, IDI, DXR, ISPD, and ISPF are known bottlenecks in the MEP pathway, and different combinations of these enzymes can yield diverse effects on terpenoid titers (Morrone et al. 2010). Therefore, we engineered strains EBTA12-15 by overexpressing IDI, DXS-IDI, DXS-DXR-IDI, and DXS-IDI-ISPD-ISDF, respectively (Table S1). The titers of T5OAc in strains EBTA12-15 were all enhanced by the reinforcement of the MEP pathway. The T5OAc titer in strain EBTA13 was higher than that of other recombinant strains, showing a 3.5-fold increase compared to EBTA11, specifically reaching 0.18 mg/L. (Fig. 2e). We believed that the possible reasons for the highest titer of T5OAc in strain EBTA13 are: (1) DXS mediates the first step to channel more metabolic flux into the MEP pathway; (2) IDI facilitates the mutual conversion of IPP and DMAPP, promoting the subsequent generation of terpenoids. In strain EBTA13, an observed accumulation of the precursor T5OH was hypothesized to result from the suboptimal catalytic efficiency of the TAT. Therefore, in order to enhance the acetylation effect of EBTA13, we replaced *Tc*TAT1 with *Tc*TAT2 from *Taxus cuspidata* and *Ca*TAT from *Corylus avellana*, resulting in the generation of strains EBTA16 and EBTA17. The titer of T5OAc in EBTA16 was 1.3-fold higher than that of EBTA13, indicating that *Tc*TAT2 facilitated the conversion of T5OH to T5OAc (Fig. 2f). However, no T5OAc was formed in EBTA17 (Fig. 2f), primarily due to the lack of *Ca*TAT expression (Supplementary Figure S4). Therefore, *Tc*TAT2 was chosen for subsequent strain modifications.

### Reducing cell metabolic burden by optimizing recombinant strain construction system

**Fig. 3:**
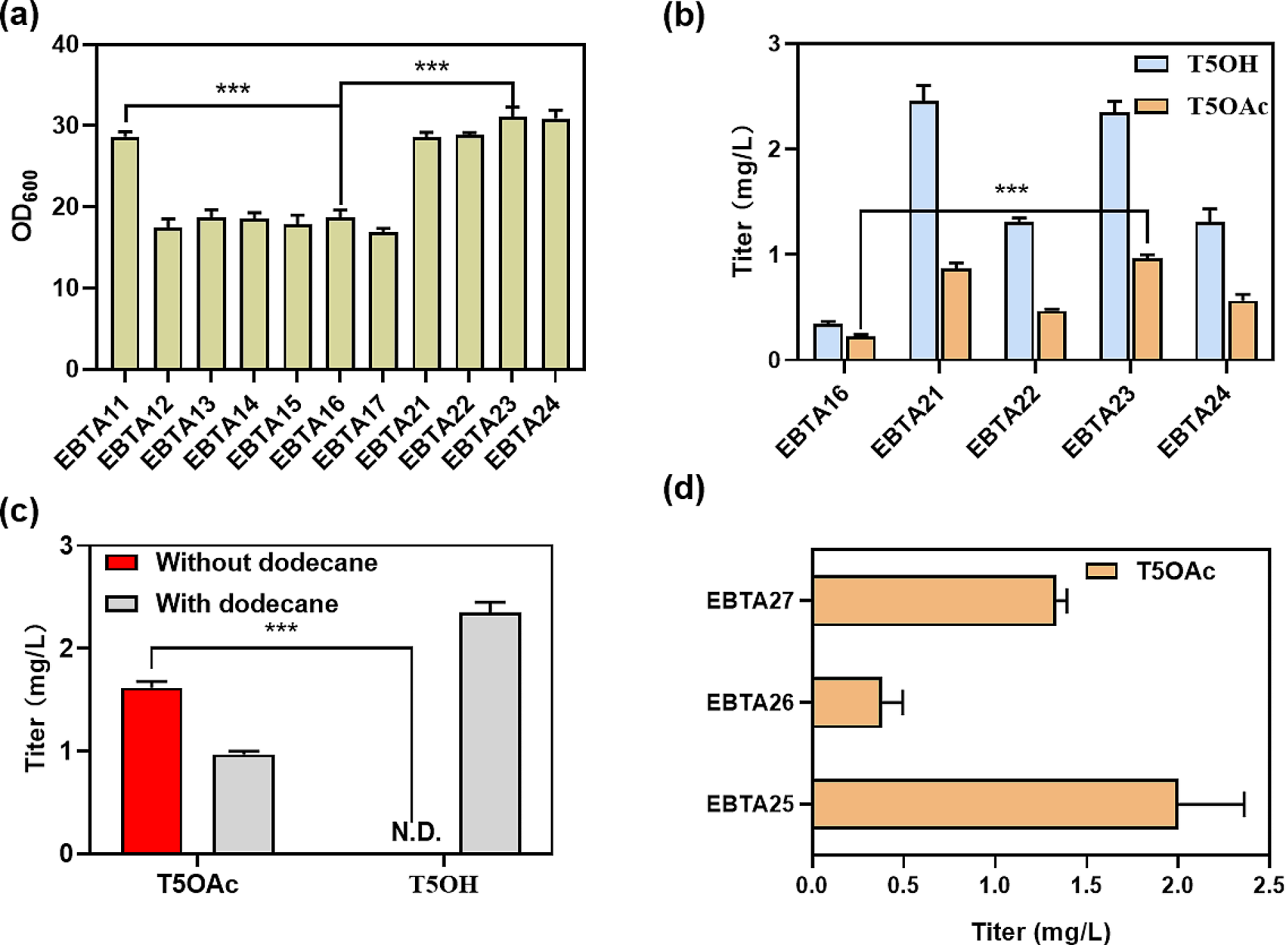
Enhancement of T5OAc production was achieved by mitigating the metabolic burden on the strain and meticulously optimizing the copy number of the plasmid. **(a)** Comparison of growth of parent strain (EBTA11), MEP pathway enhanced strains (EBTA12-17) and lower metabolic stress strains (EBTA21-24). **(b)** The plasmid production system (quantity and copy number) was optimized to obtain the strain EBTA21, EBTA22, EBTA23, EBTA24, respectively. **(c)** Differences in product distribution by culture methods. **(d)** Effect of TAT expression level on the titer of recombinant strains. The experiments were conducted in triplicate, and the results are presented with error bars representing standard deviations. Significance (*p*-value) was evaluated by two-sided t-test, *** presents *p* < 0.01. N.D., not detected

The presence of multiple plasmids in the strain may increase the metabolic burden and inhibit growth (Wu et al. 2022). We observed that all three-plasmid strains exhibited poor growth, with an OD_600_ of only 18.7 (Fig. 3a). Therefore, we attempted new construction methods to adjust the number of plasmids in the strain. Additionally, we took the plasmid copy number factor into account, and pATGTCT2 (p15A Origin, low copy plasmid) or pRTGTCT2 (RSF Origin, high copy plasmid) were combined with p40T7-DXS-IDI to construct dual-plasmid strains EBTA21 and EBTA22, respectively (Table S1). Compared with the three-plasmid strain EBTA16, the OD_600_ values of EBTA21-22 were significantly improved to 28.6 and 28.9, and the titers of T5OAc were increased to 0.87 and 0.47 mg/L, representing 3.8- and 2.0-fold increases compared to EBTA16, respectively (Fig. 3a and b). Subsequently, single-plasmid strains EBTA23 with low copy plasmid pADITGTCT2 and EBTA24 with high copy plasmid pRDITGTCT2 were generated (Table S1). Strain EBTA23 exhibited an OD_600_ of 31.1 and a titer 1.1-fold that of EBTA21 at 0.97 mg/L (Fig. 3a and b). However, the replacement of high copy plasmid reduced the production, from 0.97 to 0.56 mg/L (Fig. 3b).

Compared with strain EBTA16, strain EBTA23 exhibited a higher accumulation of the precursor T5OH (T5OH: T5OAc = 1.5:1.0, 2.4:1.0). We hypothesized that the reasons were: (1) T5OH was extracted into the *n*-dodecane phase and cannot be rapidly catalyzed by TAT and (2) the expression level of key enzyme TAT was insufficient. The accumulation of T5OH was eliminated without *n*-dodecane, and the titer of T5OAc increased by 65%, from 0.97 to 1.6 mg/L (Fig. 3c). It should be noted that in the biphasic fermentation experiments, we also measured the intracellular contents. The titer for T5OAc was determined to be 0.18 mg/L, while T5OH was not detected (Supplementary Figure S5). The suitable expression level of TAT was investigated, based on strain EBTA23, recombinant strains EBTA25, EBTA26 and EBTA27 were constructed with the general plasmid pADITGTC and the TAT regulatory plasmids pRTrcT2, pCTacT2 and pTT7T2 (TAT expression level increased gradually), respectively (Table S1). The T5OAc titer of strain EBTA25 increased by 25% compared to EBTA23, reaching 2.0 mg/L, while the titers of T5OAc in EBTA26 and EBTA27 were significantly reduced (Fig. 3d). This suggested that the moderate expression of TAT was crucial for the production of T5OAc.

### Balance of metabolic flux by multivariate-modular metabolic engineering

**Fig. 4:**
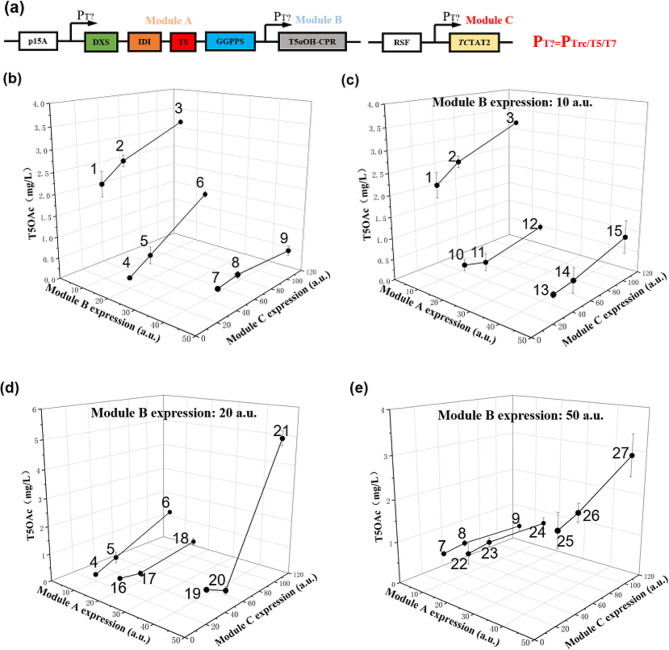
Improved production of T5OAc by multivariate-modular metabolic engineering. **(a)** The metabolic model is composed of the modules for synthesis, oxidation and acetylation of Tax. The titer of T5OAc of first, second and third round **(b)**, fourth round **(c, d, e)** modular metabolic engineering. The experiments were conducted in triplicate, and the results are presented with error bars representing standard deviations

The synthetic efficiency of the target products is affected by the metabolic flux, and intermediates accumulation may occur when the flux is imbalanced (Lim et al. 2016; Glasscock et al. 2021). Multivariate-modular metabolic engineering modularizes metabolic pathways and adjusts the expression of each module, thereby improving metabolic efficiency and ensuring that the flux is efficiently directed toward the target product (Wei et al. 2018; Liu et al. 2015; Zhang et al. 2022; Guo et al. 2022). Our study revealed that even marginal alterations in the expression levels of the TAT profoundly influenced the biosynthesis of T5OAc (Fig. 3d), underscoring the need for precise adjustment of TAT expression levels. Consequently, we hypothesized that the expression levels of other pathway enzymes also influenced the production of T5OAc. In order to achieve a balance in metabolic flux, we divided the T5OAc biosynthesis pathway into three modules. The first module (Module A) comprised DXS-IDI-TS-GGPPS, responsible for synthesized Tax from glycerol. The second module (Module B) consisted of the fusion protein T5αOH-CPR, which produced T5OH from Tax. The third module (Module C) was formed by TcTAT2, which converted T5OH into T5OAc (Fig. 4a).

To achieve full oxidation and acetylation of Tax, we meticulously optimized the expression levels of Module B and Module C while keeping the expression level of Module A constant. Initially, the expression level of Module C was increased from 20 a.u. to 100 a.u., while Modules A and B maintained a constant expression level of 10 a.u. during the first round of engineering (strains 1–3, Table S1). The findings of the study indicated that an elevation in the expression level of Module C, the titer of T5OAc also increased, rose from 2.2 to 3.3 mg/L, represented 65% increase compared to EBTA25 (Fig. 4b). Additionally, the production of Tax and by-products also increased (Supplementary Figures S6-S7). However, the fact T5OH was not detected indicated that the primary factor for improving T5OAc production under these conditions was enhancing the supply of T5OH. Consequently, during the second (20 a.u.) and third rounds (50 a.u.) of modular engineering, we escalated the expression level of Module B. This strategic enhancement, applied to strains 4–6 and 7–9 (Table S1), was aimed at facilitating the conversion of the accumulated Tax into T5OH. Unfortunately, this led to a decrease in the production of taxanes (Fig. 4b, Supplementary Figures S6-S7). Cause may be due to the change in module B that disrupted the original metabolic state, which resulted in a disorderly change on the titer of taxanes. This phenomenon was not rare. In the experiment of regulating the expression of CYP725A4, Biggs et al. found that the expression level of CYP725A4 exceeded 9 a.u. As a result, the titers of Tax and T5OH dropped sharply. Furthermore, with the increase of CYP725A4 expression level, the titer fluctuated up and down. (Biggs et al. 2016).

After three rounds of modular engineering, we obtained the optimal production strain 3, where the expression levels between modules were 10, 10, and 100 a.u., respectively. Under these conditions, the titer of T5OAc reached 3.3 mg/L. However, this result was based on the assumption of unchanged module A expression levels. To further explore the impact of changes in module A expression, additional investigations are required. In the fourth round of modular engineering, we varied the expression level of module A in strains 1–9 to identify the optimal metabolic balance strain (strains 10–27, Table S1). In strains 6, 18, and 21, as well as strains 9, 24, and 27, the titer of T5OAc increased as the expression level of module A increased. This indicated that when the downstream pathway had sufficiently strong expression, Tax could undergo continuous oxidation and acetylation. Remarkably, in strain 21, the titer of T5OAc reached 5.1 mg/L (Fig. 4d and e), which represented 55% increase compared to strain 3. Additionally, the accumulation of Tax in strain 21 significantly decreased compared to strain 3 (Supplementary Figures S6-S9). We believed that the metabolism reached a balanced state in strain 21 (EBTA321), the expression levels of modules A, B, and C were 50, 20, and 100 a.u., respectively.

### Scale-up production of T5OAc by EBTA321 in a 5-L bioreactor

Following the development of the optimized metabolic engineering strain EBTA321, fermentation conditions were meticulously refined in shake-flask. This refinement process involved systematic evaluations of various parameters, including the glycerol concentration, the inducer concentration, and the induction temperature. Unfortunately, there was no increase in the titer (Supplementary Figures S10-S12). The change of glycerol concentration had an effect on OD_600_ and the titer of T5OAc. The growth was better at higher concentrations of glycerol, but the titer of T5OAc was lower, which indicated that there was a trade-off between the growth of the strain and its production capacity. When the temperature exceeded 22 °C, the titer of T5OAc dropped significantly, which may be due to the adverse effects of high temperature on enzymes expression.

**Fig. 5:**
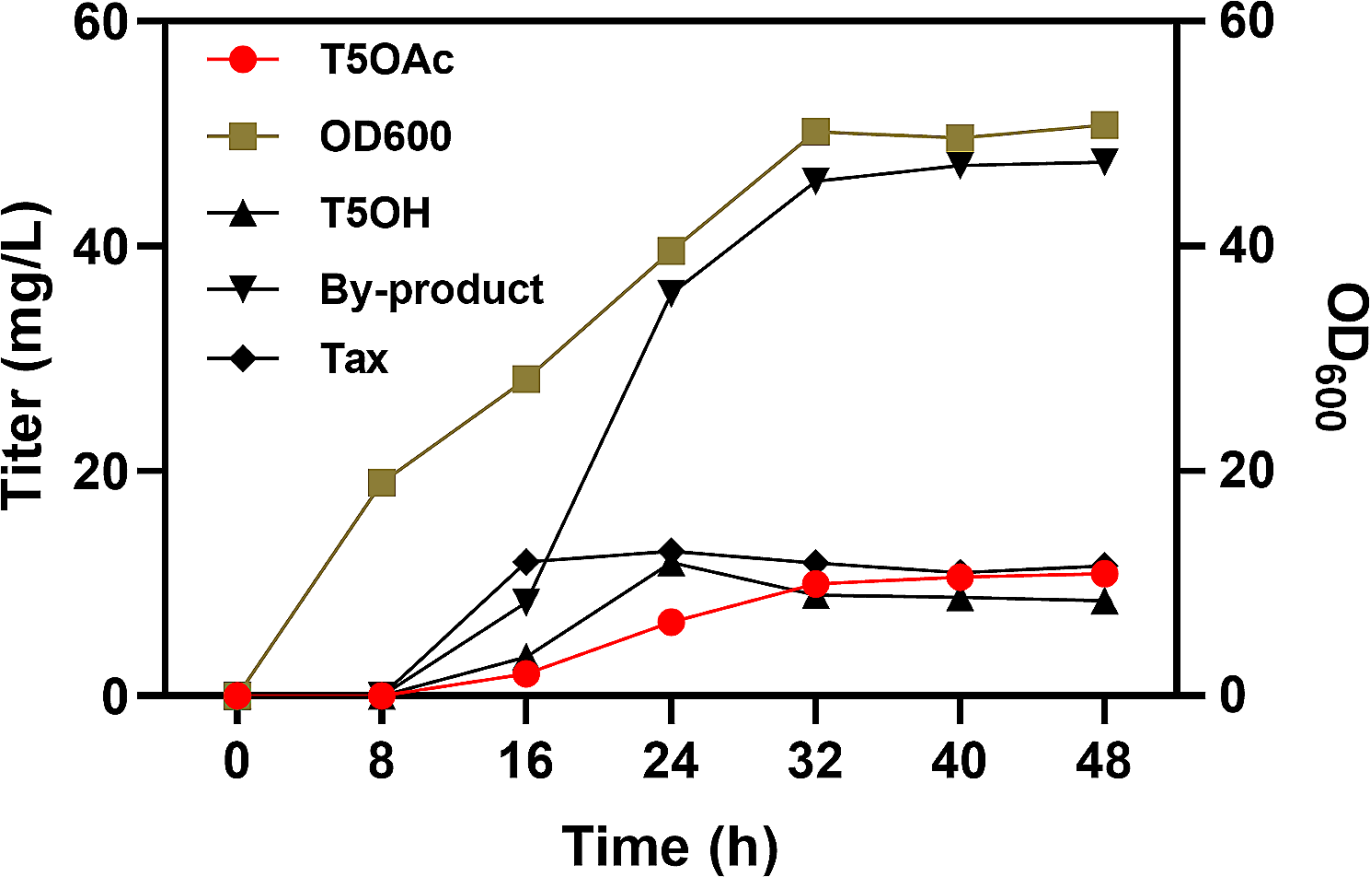
Bioreactor cultivations of strain EBTA321 were performed. In the fermentation process, DO and pH were controlled at 30% and 7.0, respectively. Feeding was controlled using a DO-Stat strategy to sustain growth. The strain was incubated at 37 °C for 4–6 h. Upon reaching an OD of 15, the temperature was lowered to 18 °C, and inducers were added to initiate the production of taxanes

Finally, the scale-up production of the strain EBTA321 was conducted in bioreactor (5 L), utilizing fed-batch culture governed by the DO-stat method. After 48 h of fermentation, we observed that the titer of T5OAc escalated to 10.9 mg/L (Fig. 5). Concurrently, the total taxanes titer achieved was 78.4 mg/L (Fig. 5). To our knowledge, these results represent the highest reported production levels of T5OAc *E. coli*. It should be mentioned that the production was scaled up, the precursor T5OH was not completely acetylated, and the accumulation phenomenon occured again, indicating that the activity of acetyltransferase may become an obstacle toward increasing the production of T5OAc and needs to be further investigated in the future.

## Discussion

In this study, we constructed recombinant strains EBTO1 and EBTO2 using the natural MEP pathway of *E. coli* and introducing heterologous genes of GGPPS, TS, and T5αOH-CPR. The copy number of plasmid was shown to affect expression of the enzymes, and further affect the titer of T5OH. Based on EBTO1, strain EBTA11 was constructed by co-expression of TAT, which yielded a T5OAc titer of 0.04 mg/L. Previous studies have indicated that overexpression of related enzymes can increase the titer of terpenoids (Das et al. 2007; Morrone et al. 2010). Therefore, we overexpressed genes of IDI, DXS-IDI, DXS-DXR-IDI and DXS-IDI-ISPD-ISPF respectively to identify the most effective enzyme combination for enhancing T5OAc production. Notably, strain EBTA13, overexpressing DXS-IDI, achieved an improved T5OAc titer of 0.18 mg/L. In heterologous biosynthesis, the catalytic performance of enzymes was affected by their sources. Therefore, we tested two TATs to improve the acetylation ability, resulting in a further increase in the T5OAc titer (up to 0.23 mg/L).

Although the production of T5OAc was improved after the enhancement of MEP pathway, growth of the strain was inhibited, which might be caused due to the high number of plasmids (Biggs et al. 2016; Hao et al. 2021). Therefore, double-plasmid and single-plasmid strains were constructed, and low-copy and high-copy plasmids were used to optimize the production. As a result, growth of the optimal strain (single plasmid, low copy) was restored significantly and the corresponding titer was further increased up to 0.97 mg/L. To reduce volatilization of terpenoids, *n*-dodecane was added to cover the culture broth. However, this cultivation method may make the metabolic intermediate unable to be further catalyzed due to early extraction (Hirte et al. 2018). In our experiments, the accumulation of intermediate T5OH was observed. Two methods were used to alleviate the accumulation of T5OH. First, we conducted a fermentation experiment without *n*-dodecane, the T5OAc titer was increased to 1.6 mg/L. Subsequently, through TAT regulation, we further increased the titer to 2.0 mg/L. The expression of key enzymes to an appropriate degree in metabolic pathway is of paramount importance for the production of target metabolites. Zhang et al. conducted regulatory experiments on the key enzymes, HemD and HemF, during the biosynthesis of heme and observed that varying enzyme expression levels had a significant impact on the heme production (Zhang et al. 2015).

The heterologous biosynthesis efficiency of microorganisms is affected by the expression level of enzymes, and the coordinated expression of enzymes is crucial for regulating the flux toward the end product. The multivariate-modular metabolic engineering approach can regulate the metabolic pathway in the limited combination space, this approach was designed to establish metabolic balance and thereby augment the biosynthesis of the desired target products (Vogl et al. 2016; Zhang et al. 2022). Wu et al. divided the resveratrol synthesis pathway into coumaroyl-CoA, malonyl-CoA and resveratrol modules, each regulated by specific plasmids and promoters. Through modular engineering, they alleviated intermediate accumulation and increased the resveratrol titer from 0.09 to 35.0 mg/L (Wu et al. 2013). In our study, we divided the metabolic pathway into the production modules of Tax, T5OH and T5OAc. By regulating these modules with the Trc, T5, and T7 promoters, in strain EBTA321, we attained a T5OAc titer of 5.1 mg/L. In this strain, T5OH was not detected, and Tax detected was only 6.37 mg/L, achieving a metabolic balance. When module A and module C were situated at a high expression level while module B was located at a low expression level, the observed T5OAc titer was the highest. Therefore, we considered that the key to improving the T5OAc production was firstly to enhance the expression of Tax synthesis module, so as to provide sufficient precursors for subsequent oxidation and acetylation. The acetylation module required a high expression level to pull metabolic flow, while the lower expression of oxidation module was better, which is consistent with the experiment of Biggs et al. to produce oxygenated taxanes (Biggs et al. 2016). In our study, the metabolic pathway was constructed based on plasmids, and the expression levels between modules were considered as the theoretical values. The actual expression levels may deviate due to plasmid instability. However, the primary aim of multivariate-modular metabolic engineering was to investigate the impact of inter-module variations’ trend on T5OAc production. Overall, we successfully increased the production of T5OAc under limited combination conditions, and further increased the production by expanding the control range of multivariate-modular metabolic engineering and by integrating enzymes into the genome to reduce metabolic stress in subsequent studies. In addition, we selected three representative strains for SDS-PAGE analysis to determine the protein expression. Overall, protein expression was observed to increase progressively with the enhancement of the promoter regulatory intensity. However, due to the expression of a wide variety of enzymes, predominantly of plant origin, some enzymes exhibited very weak expression (Supplementary Figure S13).

Only T5OH among oxygenated taxanes can be catalyzed by TAT, but the current research showed that the proportion of T5OH to the oxygenated products was between 0 and 25%, which might be affected by substrate, extraction method and culture condition (Edgar et al. 2016). So it was particularly important for the selective modification of Tax by CYP725A4, which can further enhance the accumulation of T5OH to provide sufficient precursor for the introduction of subsequent pathways, but the progress has so far been very limited (Yadav 2014).

When *S. cerevisiae* was used as the host, T5OAc was not detected in the shake-flask, only 3.7 mg/L was accumulated in the bioreactor (Walls et al. 2020). Subsequently, the titer was further increased to 22 mg/L through high-throughput screening of fermentation parameters and enhancement of strain adaptability (Walls et al. 2022). In addition, T5OAc titers reached 95 mg/L after 10 days of semi-continuous cultivation by employing three different resins for the specific adsorption of taxanes (Santoyo-Garcia et al. 2023). Therefore, in the future research, we may also improve the T5OAc production through the same strategy. Furthermore, we also detected the acetylation product of geranylgeraniol, geranylgeranyl acetate, indicating TAT exhibited non-specific catalytic activity (Walker et al. 1999). Meanwhile, two substances with the same molecular weight as T5OAc were observed (Supplementary Figures S14-S15), suggesting that the substrate of TAT may not be limited to T5OH only. These may also be isomers of T5OAc, although the specific reason requires further investigation. However, attempts to identify their structures failed because the desired separation and purification of those compounds were very difficult due to their extremely low concentrations.

In conclusion, our study effectively established a highly efficient *E. coli*-based cell factory for the synthesis of T5OAc. The production of T5OAc was effectively increased by both enhancing the natural MEP pathway and alleviating the metabolic burden on the engineered strain. Multivariate-modular metabolic engineering approach was adopted to balance the metabolic flux, resulting in T5OAc titers of 5.1 and 10.9 mg/L in shake-flask and bioreactor, respectively. The various metabolic and fermentation engineering strategies reported in this study to increase the titer of T5OAc can provide important references for increasing the production of terpenoids and the resultant strain will be useful for promoting the biosynthesis of paclitaxel.

### Electronic supplementary material

Below is the link to the electronic supplementary material.

Supplementary Material 1

Supplementary Material 2

Supplementary Material 3

## Data Availability

All data produced and analyzed in the course of this study have been comprehensively incorporated into this article and the accompanying supplementary information file.
