# Supplementary material for: Enhancing the biosynthesis of taxadien-5α-yl-acetate in Escherichia coli by combinatorial metabolic engineering approaches
Source: Bioresour Bioprocess. 2024 May 16;11(1):50. doi: 10.1186/s40643-024-00762-8 (PMC11098985; doi:10.1186/s40643-024-00762-8)
Supplement: Supplementary file 2 — Supplementary Material 2 [file 40643_2024_762_MOESM2_ESM.pdf]

Supporting Information

Enhancing the Biosynthesis of Taxadien-5α-yl-acetate in *Escherichia coli* by Combinatorial Metabolic Engineering Approaches

Wen-Liang Xie, Mei-Fang Zhang, Zheng-Yu Huang, Man Xu, Chun-Xiu Li, and Jian-He Xu*

Laboratory of Biocatalysis and Synthetic Biotechnology, State Key Laboratory of Bioreactor Engineering, East China University of Science and Technology, 130 Meilong Road, Shanghai 200237, P. R. China

*Correspondence should be addressed to J.-H.X.: jianhexu @ecust.edu.cn

Supplementary Tables

Table S1. Strains involved in this study.

Strain	Description
Top 10	Gene choning
BL21(DE3)	Production host
EBTO1	*E. coli* BL21(DE3) harboring pRSFDuet-1-TS-GGPPS and pACYCDuet-1-T5αOH-CPR
EBTO2	*E. coli* BL21(DE3) harboring pATG and pRTC
EBTA11	*E. coli* BL21(DE3) harboring pATCT1 and pRSFDuet-1-TS-GGPPS
EBTA12	EBTA11 harboring p40T7-IDI
EBTA13	EBTA11 harboring p40T7-DXS-IDI
EBTA14	EBTA11 harboring p40T7-DXS-DXR-IDI
EBTA15	EBTA11 harboring p40T7-DXS-IDI-ISPD-ISPF
EBTA16	*E. coli* BL21(DE3) harboring p40T7-DXS-IDI, pATCT2 and pRSFDuet-1-TS-GGPPS
EBTA17	*E. coli* BL21(DE3) harboring p40T7-DXS-IDI, pATCT3 and pRSFDuet-1-TS-GGPPS
EBTA21	*E. coli* BL21(DE3) harboring p40T7-DXS-IDI and pATGTCT2
EBTA22	*E. coli* BL21(DE3) harboring p40T7-DXS-IDI and pRTGTCT2
EBTA23	*E. coli* BL21(DE3) harboring pADITGTCT2
EBTA24	*E. coli* BL21(DE3) harboring pRDITGTCT2
EBTA25	*E. coli* BL21(DE3) harboring pADITGTC and p20TrcT2
EBTA26	*E. coli* BL21(DE3) harboring pADITGTC and pCTacT2
EBTA27	*E. coli* BL21(DE3) harboring pADITGTC and PTT7T2
EBTA31 [a]	*E. coli* BL21(DE3) harboring p10TrcDITGTrcTC and p20TrcT2
EBTA32 [a]	*E. coli* BL21(DE3) harboring p10TrcDITGTrcTC and p20T5T2
EBTA33 [a]	*E. coli* BL21(DE3) harboring p10TrcDITGTrcTC and p20T7T2
EBTA34 [a]	*E. coli* BL21(DE3) harboring p10TrcDITGT5TC and p20TrcT2
EBTA35 [a]	*E. coli* BL21(DE3) harboring p10TrcDITGT5TC and p20T5T2
EBTA36 [a]	*E. coli* BL21(DE3) harboring p10TrcDITGT5TC and p20T7T2
EBTA37 [a]	*E. coli* BL21(DE3) harboring p10TrcDITGT7TC and p20TrcT2
EBTA38 [a]	*E. coli* BL21(DE3) harboring p10TrcDITGT7TC and p20T5T2
EBTA39 [a]	*E. coli* BL21(DE3) harboring p10TrcDITGT7TC and p20T7T2
EBTA310 [a]	*E. coli* BL21(DE3) harboring p10T5DITGTrcTC and p20TrcT2
EBTA311 [a]	*E. coli* BL21(DE3) harboring p10T5DITGTrcTC and p20T5T2
EBTA312 [a]	*E. coli* BL21(DE3) harboring p10T5DITGTrcTC and p20T7T2
EBTA313 [a]	*E. coli* BL21(DE3) harboring p10T7DITGTrcTC and p20TrcT2
EBTA314 [a]	*E. coli* BL21(DE3) harboring p10T7DITGTrcTC and p20T5T2
EBTA315 [a]	*E. coli* BL21(DE3) harboring p10T7DITGTrcTC and p20T7T2
EBTA316 [a]	*E. coli* BL21(DE3) harboring p10T5DITGT5TC and p20TrcT2
EBTA317 [a]	*E. coli* BL21(DE3) harboring p10T5DITGT5TC and p20T5T2
EBTA318 [a]	*E. coli* BL21(DE3) harboring p10T5DITGT5TC and p20T7T2
EBTA319 [a]	*E. coli* BL21(DE3) harboring p10T7DITGT5TC and p20TrcT2

EBTA320 [a]	*E. coli* BL21(DE3) harboring p10T7DITGT5TC and p20T5T2
EBTA321 [a]	*E. coli* BL21(DE3) harboring p10T7DITGT5TC and p20T7T2
EBTA322 [a]	*E. coli* BL21(DE3) harboring p10T5DITGT7TC and p20TrcT2
EBTA323 [a]	*E. coli* BL21(DE3) harboring p10T5DITGT7TC and p20T5T2
EBTA324 [a]	*E. coli* BL21(DE3) harboring p10T5DITGT7TC and p20T7T2
EBTA325 [a]	*E. coli* BL21(DE3) harboring p10T7DITGT7TC and p20TrcT2
EBTA326 [a]	*E. coli* BL21(DE3) harboring p10T7DITGT7TC and p20T5T2
EBTA327 [a]	*E. coli* BL21(DE3) harboring p10T7DITGT7TC and p20T7T2

[a] EBTA31-327 represents strains 1-27 of the multivariate-modular metabolic engineering.

Table S2. List of genes used in this study.

Gene	Source organism	Accession numbers
DXS	*Escherichia coli*	AF035440
IDI	*Escherichia coli*	AF119715
DXR	*Escherichia coli*	AB013300
ISPD	*Escherichia coli*	AF230736
ISPF	*Escherichia coli*	AF230738
GGPPS[a]	*Taxus canadensis*	KC686335
TS[a]	*Taxus brevifolia*	OR036887
T5αOH[a]	*Taxus canadensis*	OR036888
CPR[a]	*Taxus canadensis*	OR036889
*Tc*TAT1[a]	*Taxus cuspidata*	OR036884
*Tc*TAT2[a]	*Taxus cuspidata*	OR036885
*Ca*TAT[a]	*Corylus avellana*	OR036886

[a]Codon optimized for *E. coli*

DXR, 1-deoxy-D-xylulose 5-phosphate reductoisomerase; ISPD, 4-diphosphocytidyl-2C-methyl-D-erythritol synthase; ISPF, 2C-methyl-D-erythritol 2,4-cyclodiphosphate synthase.

Table S3 The plasmids applied and constructed in this study

Plasmid	Description (Origin of replication, Antibiotic marker, Promotors and Operons)	References
p40T7-DXS-IDI	ColE1, Amp, P_{T7}, DXS-IDI	[1]
pRSFDuet-1-TS-GGPPS	RSF, Kan, P_{T7}, TS, P_{T7}, GGPPS	[2]
pACYCDuet-1-T5αOH-CPR	p15A, Chl, P_{T7}, T5αOH-CPR	[2]
p40T7-IDI	ColE1, Amp, P_{T7}, IDI	This study
p40T7-DXS-DXR-IDI	ColE1, Amp, P_{T7}, DXS-DXR-IDI	This study
p40T7-DXS-IDI-ISPD-ISPF	ColE1, Amp, P_{T7}, DXS-IDI-ISPD-ISPF	This study
pATG	p15A, Chl, P_{T7}, TS, P_{T7}, GGPPS	This study
pRTC	RSF, Kan, P_{T7}, T5αOH-CPR	This study
pATCT1	p15A, Chl, P_{T7}, T5αOH-CPR-*Tc*TAT1	This study
pATCT2	p15A, Chl, P_{T7}, T5αOH-CPR-*Tc*TAT2	This study
pATCT3	p15A, Chl, P_{T7}, T5αOH-CPR-*Ca*TAT	This study
pATGTCT2	p15A, Chl, P_{T7}, TS-GGPPS, P_{T7}, T5αOH-CPR-*Tc*TAT2	This study
pRTGTCT2	RSF, Kan, P_{T7}, TS-GGPPS, P_{T7}, T5αOH-CPR-*Tc*TAT2	This study
pADITGTCT2	p15A, Chl, P_{T7}, DXS-IDI-TS-GGPPS, P_{T7}, T5αOH-CPR-*Tc*TAT2	This study
pRDITGTCT2	RSF, Kan, P_{T7}, DXS-IDI-TS-GGPPS, P_{T7}, T5αOH-CPR-*Tc*TAT2	This study
pADITG	p15A, Chl, P_{T7}, DXS-IDI-TS-GGPPS	This study
pADITGTC (p10T7DITGT7TC)	p15A, Chl, P_{T7}, DXS-IDI-TS-GGPPS, P_{T7}, T5αOH-CPR	This study
pRTrc	RSF, Kan, P_{trc}	This study
pRT5	RSF, Kan, P_{T5}	This study
p20TrcT2 (pRTrcT2)	RSF, Kan, P_{Trc}, *Tc*TAT2	This study
p20T5T2	RSF, Kan, P_{T5}, *Tc*TAT2	This study
p20T7T2	RSF, Kan, P_{T7}, *Tc*TAT2	This study
pCTacT2	pBR322, Amp, P_{Tac}, *Tc*TAT2	This study
pTT7T2	ColE1, Amp, P_{T7}, *Tc*TAT2	This study
pTrcHis-DITG	pBR322, Amp, P_{Trc}, DXS-IDI-TS-GGPPS	This study
pTrcHis-TC	pBR322, Amp, P_{Trc}, T5αOH-CPR	This study
p10TrcDITG	p15A, Chl, P_{Trc}, DXS-IDI-TS-GGPPS	This study
p10TrcDITGTrcTC	p15A, Chl, P_{Trc}, DXS-IDI-TS-GGPPS, P_{Trc}, T5αOH-CPR	This study
p10TrcDITGT5TC	p15A, Chl, P_{Trc}, DXS-IDI-TS-GGPPS, P_{T5}, T5αOH-CPR	This study
p10TrcDITGT7TC	p15A, Chl, P_{Trc}, DXS-IDI-TS-GGPPS, P_{T7}, T5αOH-CPR	This study
p10T5DITGTrcTC	p15A, Chl, P_{T5}, DXS-IDI-TS-GGPPS,	This study

	P_{Trc}, T5αOH-CPR	
p10T5DITGT5TC	p15A, Chl, P_{T5}, DXS-IDI-TS-GGPPS, P_{T5}, T5αOH-CPR	This study
p10T5DITGT7TC	p15A, Chl, P_{T5}, DXS-IDI-TS-GGPPS, P_{T7}, T5αOH-CPR	This study
p10T7DITGTrcTC	p15A, Chl, P_{T7}, DXS-IDI-TS-GGPPS, P_{Trc}, T5αOH-CPR	This study
p10T7DITGT5TC	p15A, Chl, P_{T7}, DXS-IDI-TS-GGPPS, P_{T5}, T5αOH-CPR	This study

Amp, Ampicillin; Kan, kanamycin; Chl, chloramphenicol.

Table S4 The primers applied and constructed in this study

Primer	Sequence (5'-3')
1F	ATGCAAACGGAACACGTCATTTTATTGAA
1R	TTATTTAAGCTGGGTAAATGCAGATAATCGTT
2F	CATTTACCCAGCTTAAATAAGCTAGCATGACTGGTG GACA
2R	ATGACGTGTTCCGTTTGCATATGTATATCTCCTTCTT AAAGTTAAACAAAATTATTTC
3F	ATGAAGCAACTCACCATTCTGGG
3R	ATGTATATCTCCTTCTCAGCTTGCGAGACGCATCAC
4F	GAAGGAGATATACATATGCAAACGGAA
4R	AGAATGGTGAGTTGCTTCATATGTATATCTCCTTCTT ATGCCAGC
5F	TACCCAGCTTAAATAAGAAGGAGATATACATATGGC AACCACTCATTTGGAT
5R	ATGTATATCTCCTTCTTATGTATTCTCCTGATGGATG GTTCGG
6F	GAAGGAGATATACATATGCGAATTGGACACGGTTTT GAC
6R	TCATTTTGTTGCCTTAATGAGTAGCGC
7F	ATTAAGGCAACAAAATGAGCTAGCATGACTGGTGG AC
7R	TTATTTAAGCTGGGTAAATGCAGATAATCG
8F	ATGAGCAGCAGCACCGGC
8R	TTAAACCTGAATCGGATCGATGTACACTTT
9F	ATGTTTGATTTCAATGAATATATGAAAAGTAAGGCT
9R	TCACAACTGACGAAACGCAATGTAAT
10F	TCGATCCGATTCAGGTTTAAGGCAGCAGCCATCACC AT
10R	TATTCATTGAAATCAAACATATGTATATCTCCTTCTT ATACTTAACTAATATACTAAG
11F	ATTGCGTTTCGTCAGTTGTGAGCAGATCTCAATTGGA TATCGG
11R	GTGCCGGTGCTGCTGCTCATGGTATATCTCCTTATTAA AGTTAAACAAAATT
12F	ATGGCGCTGCTGCTGGC
12R	TTACCAGATGTCACGCAGGTAACGA
13F	TTACCTGCGTGACATCTGGTAAGGCAGCAGCCATCAC CA
13R	ACCGCCAGCAGCAGCGCCATGGTATATCTCCTTATTA AAGTTAAACAAAATTATTTC
14F	GCGTGACATCTGGTAAGAAGGAGATATACATATGGAA AAAACAGATTTACACGTAAATCT
14R	TTACACCTTCGCCACGTATTTTTT

15F	CCTGCGTGACATCTGGTAAGAAGGAGATATACATATG GAACACGCTGTATGGAAA
15R	TTAAACTGCCGGAACGTATTTGTTAAT
16F	GCGTGACATCTGGTAAGAAGGAGATATACATATGGAA AAAGTATCAAATAAGAGTTGTCA
16R	TTAAATCTGCATCAGCTGCTCAAA
17F	ATACGTGGCGAAGGTGTAAGGCAGCAGCCATCACCA TCA
17R	TTACCAGATGTCACGCAGGTAAC
18F	AATACGTTCCGGCAGTTTAAGGCAGCAGCCATCACCA TC
18R	TTACCAGATGTCACGCAGGTAACGAC
19F	GCAGCTGATGCAGATTTAAGGCAGCAGCCATCACCA TCA
19R	TTACCAGATGTCACGCAGGTAAC
20F	GAAGGAGATATACATATGTTTGATTTCAATGAATATAT GAAAAGTAAGGC
20R	TCACAACTGACGAAACGCAATG
21F	TTGCGTTTCGTCAGTTGTGAGGCAGCAGCCATCACC A
21R	ACCGCCAGCAGCAGCGCCATATGTATATCTCCTTCTTA TACTTAACTAATATACTAAGAT
22F	ATGGCGCTGCTGCTGGC
22R	TTAAACTGCCGGAACGTATTTGTTAATCATGTC
23F	ATACGTTCCGGCAGTTTAAGCAGATCTCAATTGGATA TCGG
23R	CATATGTATATCTCCTTCTTAAACCTGAATCGGATCGA TGT
24F	ATGAGCAGCAGCACCGGC
24R	TTAAACTGCCGGAACGTATTTGTTAATCAT
25F	AATACGTTCCGGCAGTTTAAGGCAGCAGCCATCACC A
25R	GCCGGTGCTGCTGCTCATGGTATATCTCCTTATTAAAG TTAAACAAAATTATT
26F	ATGAGTTTTGATATTGCCAAATACCC
26R	GGTGCTGCTGCTCATATGTATATCTCCTTCTTATTTAA GCTGGGTAAATGCAGATAATCG
27F	ATGAGTTTTGATATTGCCAAATACCCGAC
27R	TTATTTAAGCTGGGTAAATGCAGATAATCGTTTTC
28F	ATGAGCAGCAGCACCGG
28R	TTGGCAATATCAAAACTCATGGTATATCTCCTTATTAA AGTTAAACAAAATTATTTC
29F	ATTTACCCAGCTTAAATAAGAAGGAGATATACATATG AGCAGCAGCACCGG

29R	TGGCAATATCAAAACTCATGGTATATCTCCTTATTAAAGTTAAACAAAATTAT
30F	ATGAGTTTTGATATTGCCAAATACCCGAC
30R	GGTGCTGCTGCTCATATGTATATCTCCTTCTTATTTAAGCTGGGTAAATGCAGATAATCG
31F	ATGAGCAGCAGCACCGGC
31R	TCACAACTGACGAAACGCAATGT
32F	TTGCGTTTCGTCAGTTGTGAGGCAGCAGCCATCACCATC
32R	TTGGCAATATCAAAACTCATGGTATATCTCCTTATTAAAGTTAAACAAAAT
33F	ATGGCGCTGCTGCTGGC
33R	TTACCAGATGTCACGCAGGTAACGA
34F	CCTGCGTGACATCTGGTAAGCAGATCTCAATTGGATATCGG
34R	GCCAGCAGCAGCGCCATATGTATATCTCCTTCTTATACTTAACTAATATACTAAG
35F	GCACTCCCGTTCTGGATAAT
35R	AAAAGGCCATCCGTCAGGAT
36F	CTCGAGAAATCATAAAAAATTTATTTGCTTT
36R	GTGATGGTGATGGTGATGCG
37F	ATCCTGACGGATGGCCTTTTCTGAAACCTCAGGCATTTGAG
37R	CCAGAACGGGAGTGCATTTCCTAATGCAGGAGTCGC
38F	TCGCATCACCATCACCATCAC
38R	ATTTTTTATGATTTCTCGAGATTTCCTAATGCAGGAGTCGCA
39F	TAAATAAGGAGGAATAAACCATGGAACACGCTGTATGGAAA
39R	GCAGATCTCGAGCTCGGATCTTAAACTGCCGGAACGTATTTGT
40F	TTAAAGAGGAGAAATTAACTATGGAACACGCTGTATGGAAA
40R	TGGTGATGCGATCCTCTTTAAACTGCCGGAACGTATTTG
41F	ACTTTAATAAGGAGATATACCATGGAACACGCTGTATGGAA
41R	GATGGTGATGGCTGCTGCCTTAAACTGCCGGAACGTATTTGTT
42F	GATCCGAGCTCGAGATCTG
42R	CATGGTTTATTCCTCCTTATTTAATCGAT
43F	AGAGGATCGCATCACCATC
43R	CATAGTTAATTTCTCCTCTTTAATGAATTCTGTG
44F	GGCAGCAGCCATCACCATC

44R	CATGGTATATCTCCTTATTAAAGTTAAACAAAATTATTT
45F	GATGCTTAGGAGGTCATATGGAACACGCTGTATGGAAAGA
45R	GCGTACTATGGTTGCTTTGATTAAACTGCCGGAACGTATTTGT
46F	ACTTTAATAAGGAGATATACCATGGAACACGCTGTATGGAA
46R	GATGGTGATGGCTGCTGCCTTAAACTGCCGGAACGTATTTGTT
47F	TCAAAGCAACCATAGTACGCGC
47R	CATATGACCTCCTAAGCATCGATGGAT
48F	GGCAGCAGCCATCACCATC
48R	CATGGTATATCTCCTTATTAAAGTTAAACAAAATTATTT
49F	ATGAGTTTTGATATTGCCAAATACCCG
49R	TCACAACTGACGAAACGCAATGTAA
50F	ATGGCGCTGCTGCTGGC
50R	TTACCAGATGTCACGCAGGTAACGACC
51F	TTGCGTTTCGTCAGTTGTGAGATCCGAGCTCGAGATCTG
51R	TTGGCAATATCAAAACTCATGGTTTATTCCTCCTTATTTAATCGAT
52F	ACCTGCGTGACATCTGGTAAGATCCGAGCTCGAGATCTG
52R	ACCGCCAGCAGCAGCGCCATGGTTTATTCCTCCTTATTTAATCGATACA
53F	AATGAGCTGTTGACAATTAATCATCCG
53R	AAAAGGCCATCCGTCAGGAT
54F	ATCCTGACGGATGGCCTTTTGGCAGCAGCCATCACCATCAT
54R	TTAATTGTCAACAGCTCATTATTTCCTAATGCAGGAGTCGCAT
55F	ATGCTTCTGGCGTCAGGCAG
55R	TCAAATGCCTGAGGTTTCAGAAAAGGCCATCCGTCAGGATGG
56F	CTGAAACCTCAGGCATTTGAGAAG
56R	CTGCCTGACGCCAGAAGCATTTATGCGGCCGCAAGCTTG

Supplementary Figures

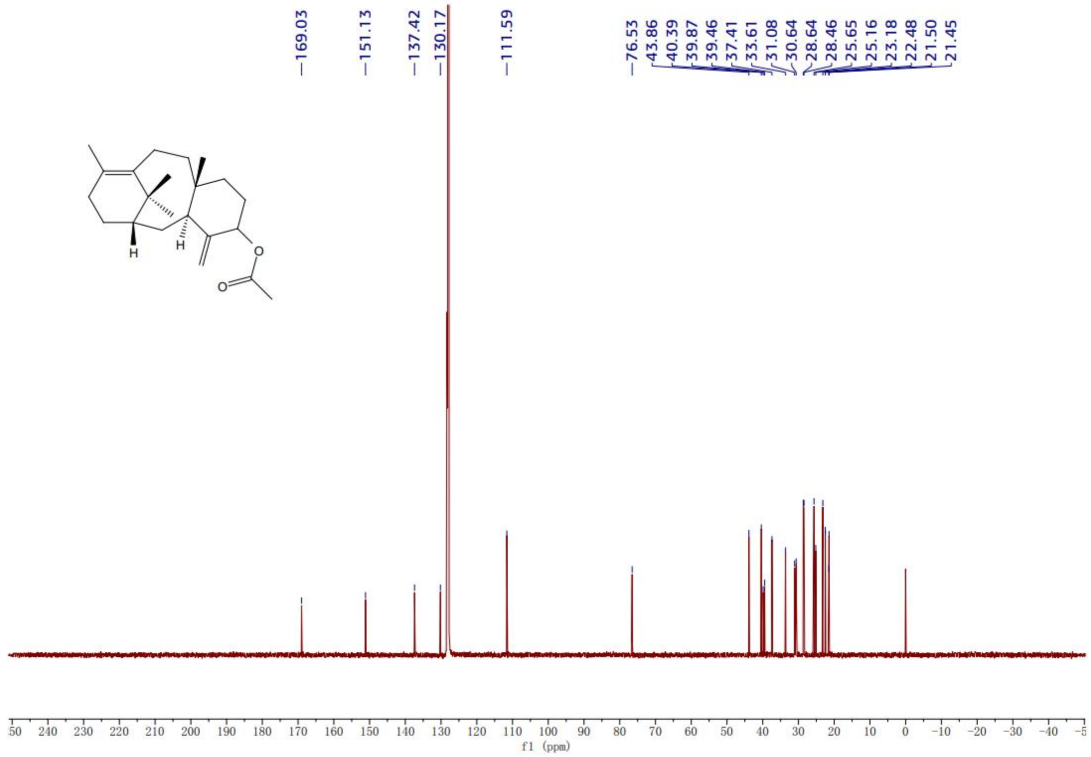

Supplementary Figure 1: **13C NMR** of taxadien-5α-yl-acetate. **13C NMR** (151 MHz, C6D6) δ 169.03, 151.13, 137.42, 130.17, 111.59, 76.53, 43.86, 40.39, 39.87, 39.46, 37.41, 33.61, 31.08, 30.64, 28.64, 28.46, 25.65, 25.16, 23.18, 22.48, 21.50, 21.45.

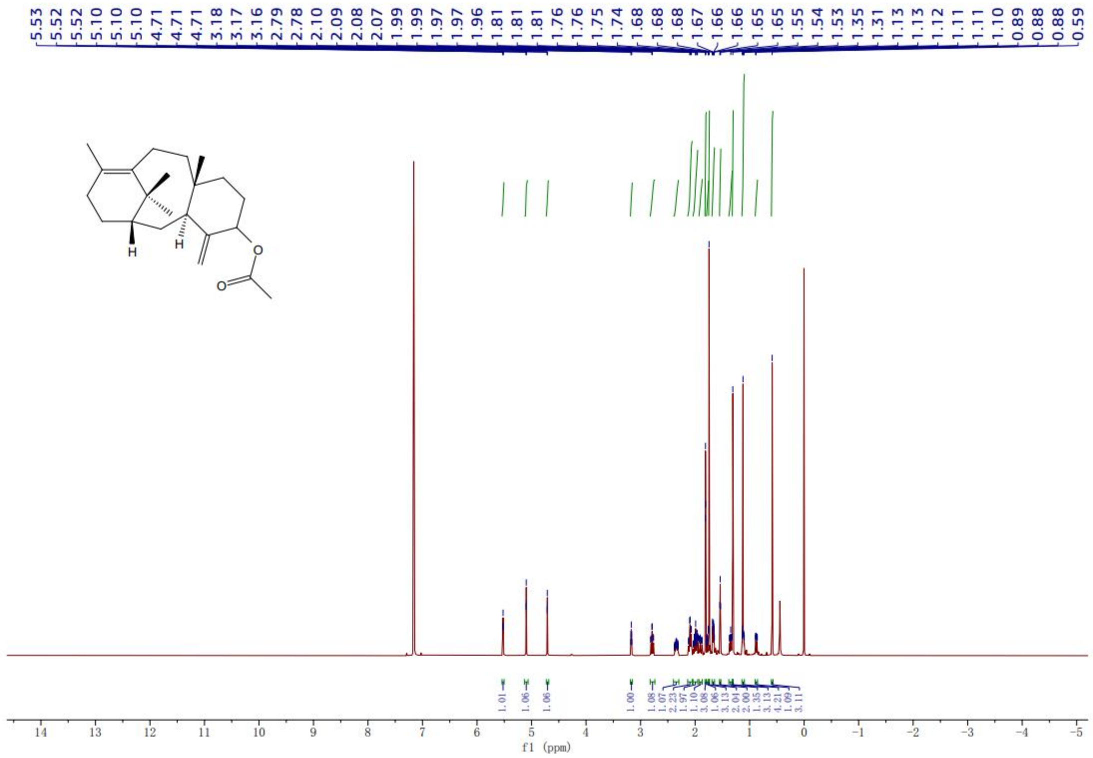

Supplementary Figure 2: **1H NMR** of taxadien-5α-yl-acetate. **1H NMR** (600 MHz, C6D6) δ 5.52 (s, 1H), 5.10 (s, 1H), 4.71 (s, 1H), 3.17 (s, 1H), 2.79 (td, J = 13.6, 5.3 Hz, 1H), 2.40 – 2.30 (m, 1H), 2.10 (td, J = 13.6, 4.7 Hz, 2H), 2.04 – 1.95 (m, 2H), 1.93 – 1.87 (m, 1H), 1.81 (s, 3H), 1.79 – 1.75 (m, 1H), 1.74 (s, 3H), 1.67 (ddd, J = 14.3, 5.4, 3.9 Hz, 2H), 1.54 (t, J = 3.9 Hz, 2H), 1.35 (ddd, J = 14.9, 10.3, 5.0 Hz, 1H), 1.31 (s, 3H), 1.14 – 1.10 (m, 4H), 0.90 – 0.85 (m, 1H), 0.59 (s, 3H).

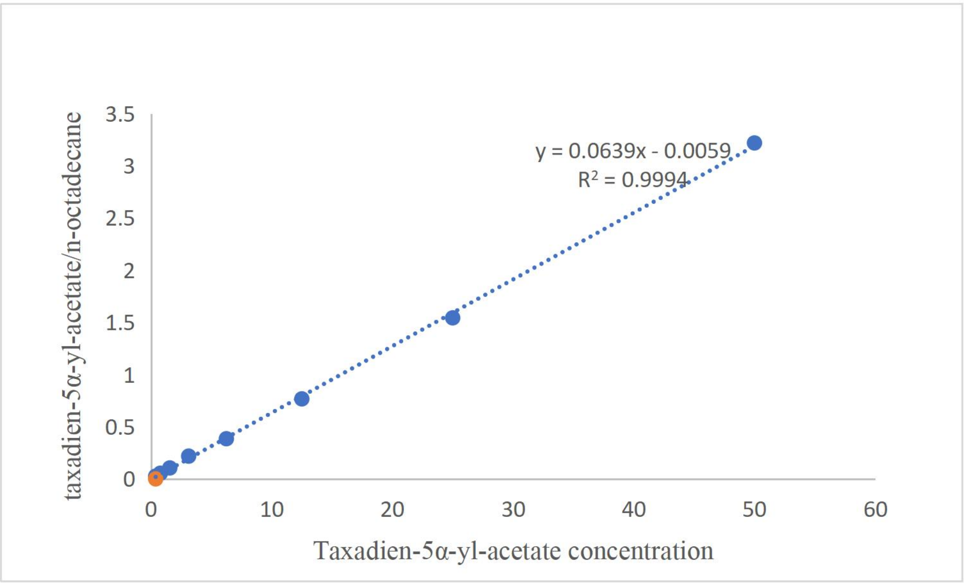

Supplementary Figure 3: Calibration curve for taxadien-5α-yl-acetate/*n*-octadecane and taxadien-5α-yl-acetate concentration.

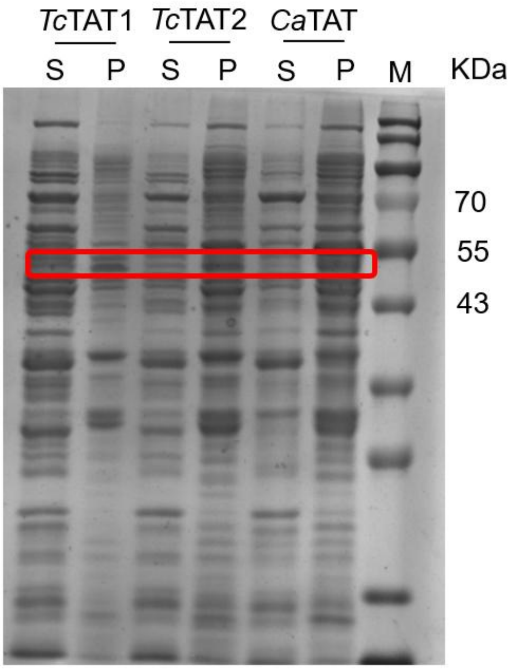

Supplementary Figure 4: Inducible expression of TAT at 18 °C. S: supernatant; P: precipitation.

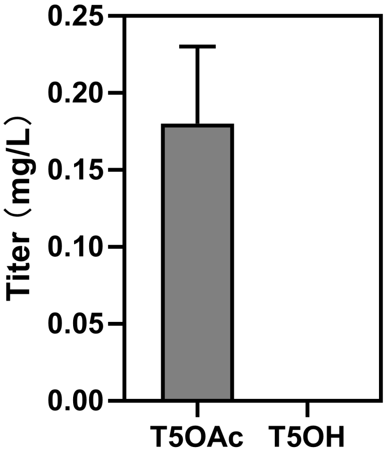

Supplementary Figure 5: Intracellular products distribution of strain EBTA23 with *n*-dodecane.

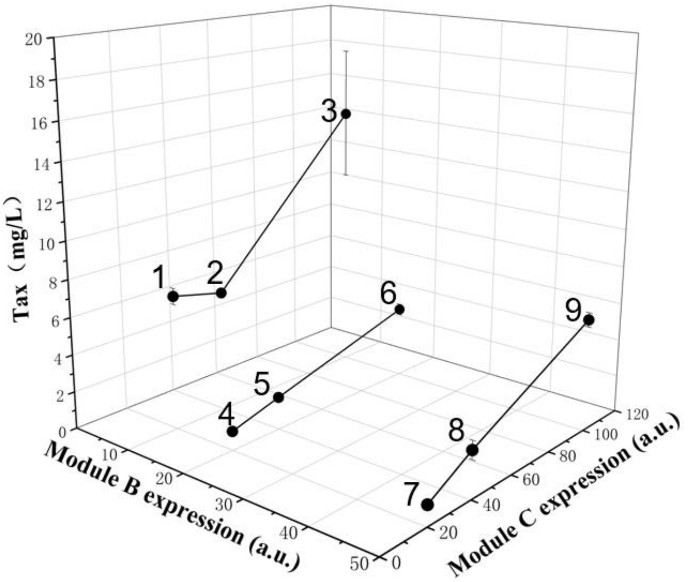

Supplementary Figure 6: The titer of Tax in modular metabolic engineering strains 1-9.

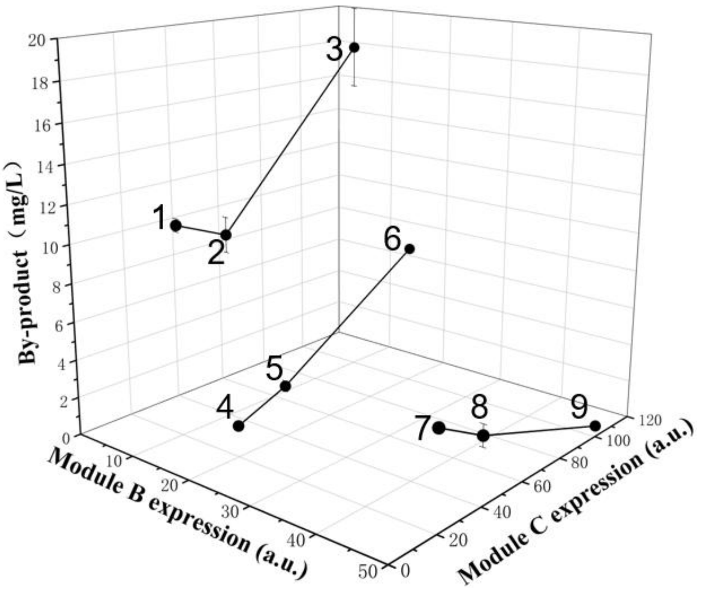

Supplementary Figure 7: The titer of By-products (except for T5OH in the oxygenated products) in modular metabolic engineering strains 1-9.

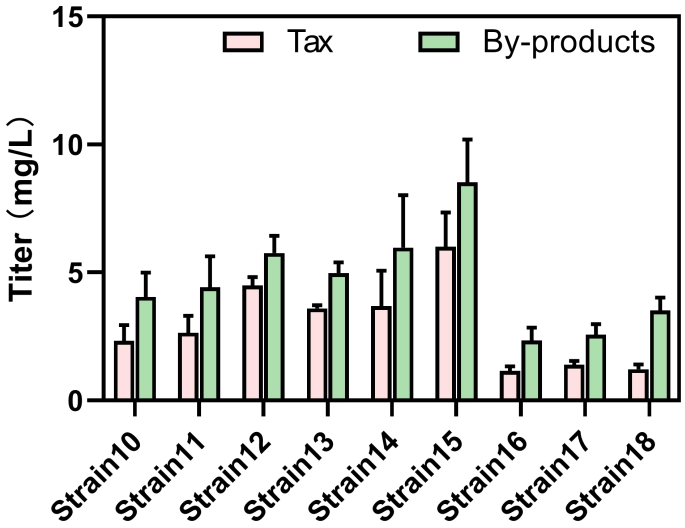

Supplementary Figure 8: The titer of Tax and By-products (except for T5OH in the oxygenated products) in modular metabolic engineering strains 10-18.

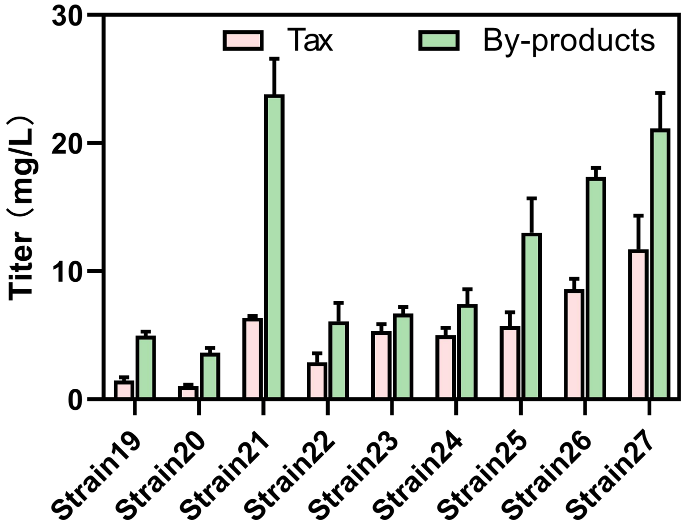

Supplementary Figure 9: The titer of Tax and By-products (except for T5OH in the oxygenated products) in modular metabolic engineering strains 19-27.

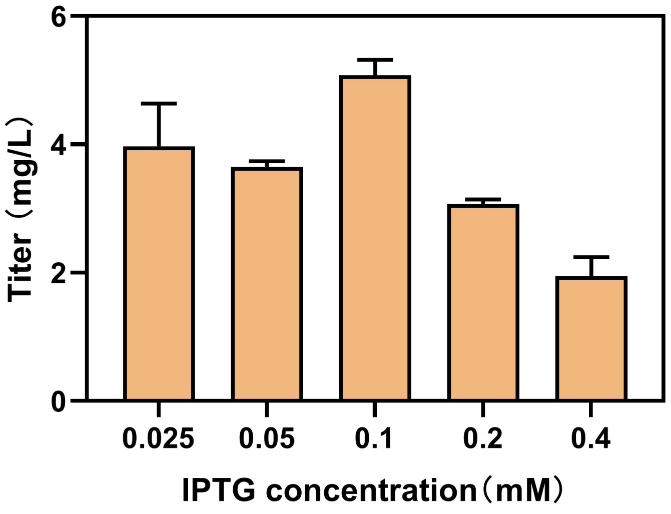

Supplementary Figure 10: Optimization of T5OAc titer in strain EBTA321 through the inducer concentration.

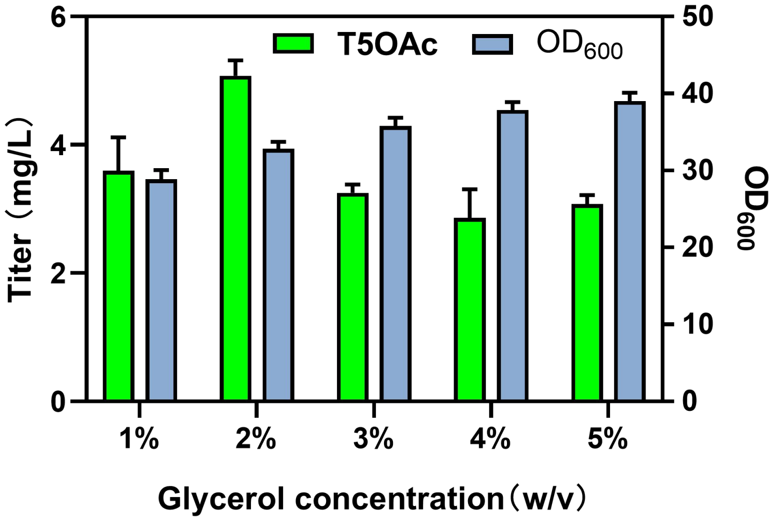

Supplementary Figure 11: Optimization of the glycerol concentration for EBTA321 strain.

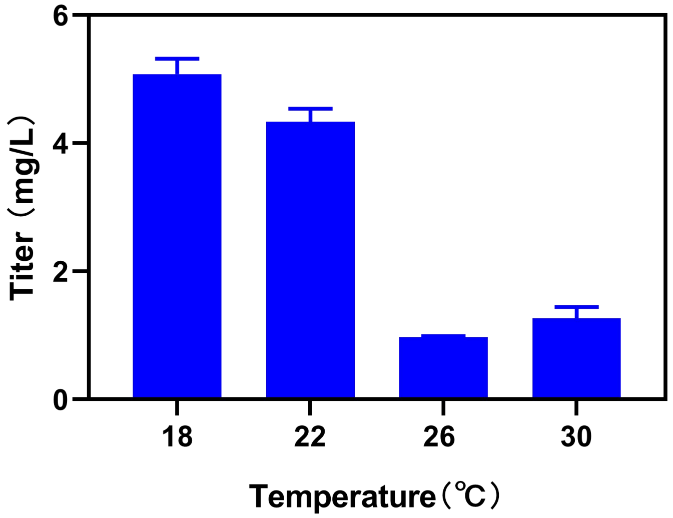

Supplementary Figure 12: Optimization of T5OAc titer in strain EBTA321 through the temperature.

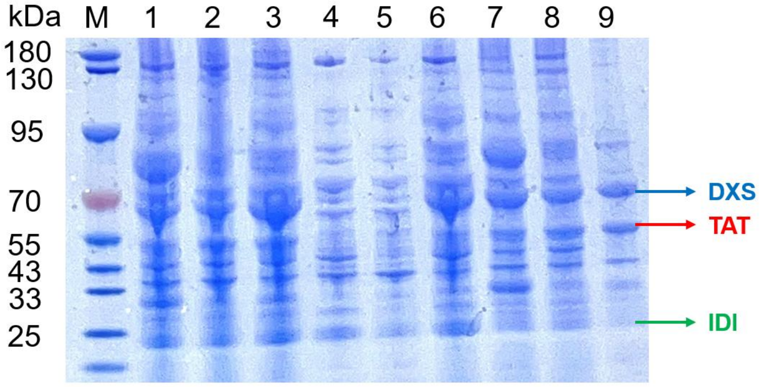

Supplementary Figure 13: SDS-PAGE analysis of pathway enzymes of Multivariate-Modular Metabolic Engineering in *E. coli*. M: marker; Lane M: protein marker; Lane 1-3: Whole cell of EBTA31, EBTA317, EBTA327; Lane 4-6: Supernatant of EBTA31, EBTA317, EBTA327; Lane 7-9: Precipitation of EBTA31, EBTA317, EBTA327.

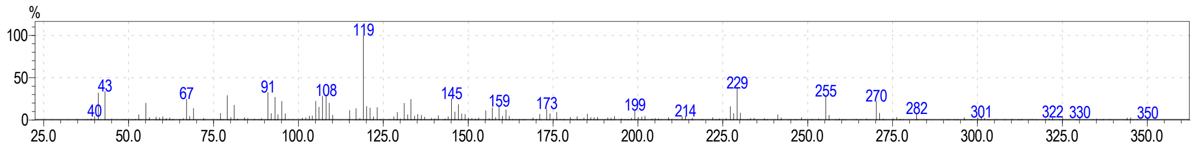

Supplementary Figure 14: Unknown compound 1 mass spectrum.

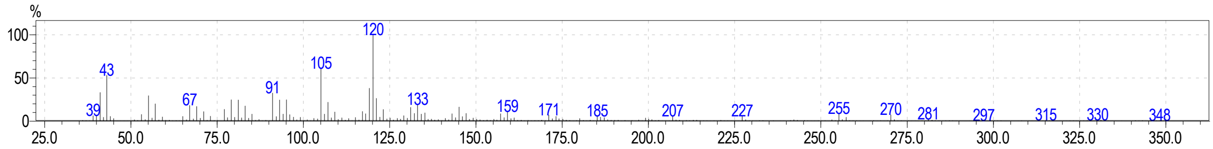

Supplementary Figure 15: Unknown compound 2 mass spectrum.

References

(1) Du FL, Yu HL, Xu JH, Li CX (2014) Enhanced limonene production by optimizing the expression of limonene biosynthesis and MEP pathway genes in *E coli*. *Bioresour Bioprocess 1* (1): 10. Doi: 10.1186/s40643-014-0010-z

(2) Wu QY, Huang ZY, Wang JY, Yu HL, Xu JH (2022) Construction of an *Escherichia coli* cell factory to synthesize taxadien-5 alpha-ol, the key precursor of anti-cancer drug paclitaxel. *Bioresour Bioprocess 9* (1): 82. Doi: 10.1186/s40643-022-00569-5
