# Supplementary figures and images for: Enhancing the biosynthesis of taxadien-5α-yl-acetate in Escherichia coli by combinatorial metabolic engineering approaches
Source: Bioresour Bioprocess. 2024 May 16;11(1):50. doi: 10.1186/s40643-024-00762-8 (PMC11098985; doi:10.1186/s40643-024-00762-8)

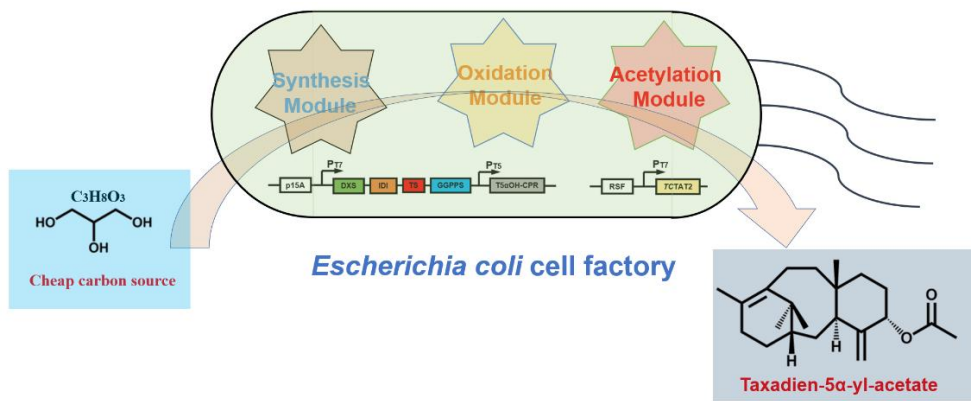

Supplement: Supplementary file 3 — Supplementary Material 3 [file 40643_2024_762_MOESM3_ESM.pdf]
