# Supplementary material for: Enhancing the biosynthesis of taxadien-5α-yl-acetate in Escherichia coli by combinatorial metabolic engineering approaches
Source: Bioresour Bioprocess. 2024 May 16;11(1):50. doi: 10.1186/s40643-024-00762-8 (PMC11098985; doi:10.1186/s40643-024-00762-8)
Supplement: Supplementary file 1 — Supplementary Material 1 [file 40643_2024_762_MOESM1_ESM.docx]

Dear editor and reviewers

We are grateful for your review and the constructive suggestions provided for our manuscript titled ‘Enhancing the Biosynthesis of Taxadien‐5α‐yl‐acetate in *Escherichia coli* by Combinatorial Metabolic Engineering Approaches’ (BIOB-D-23-00207). These suggestions are very useful for improving the quality of our manuscript, and also have great guiding significance for our subsequent research. After careful discussion, we have made corresponding modifications to the manuscript based on your suggestions and hope to receive your approval. The modified parts are marked in the manuscript. The following are detailed responses to the reviewer's comments:

Point-to-point responses

Reviewer #1: This study reports the production of T5OAc in *E. coli*, achieving a yield of 10.9 mg/L in a 5L bioreactor through pathway reconstruction and optimization. The manuscript presents robust data; however, certain aspects require further attention for publication:

1. Revision of Introduction: The introduction states that T5OAc has been produced at levels of 26 mg/L and 95 mg/L with resin. The authors are advised to clarify their rationale for continuing T5OAc production in *E. coli*, given these existing production levels.

Response: The introduction section of our manuscript was further supplemented with a rationale for employing *E. coli* for the production of T5OAc. Currently, the highest titers of T5OAc have been obtained using the *S. cerevisiae* LR6 strain. This strain achieved T5OAc levels of 26 mg/L through *in silico* design and 95 mg/L using a ten-day semi-continuous cultivation combined with combinatorial resin adsorption (Malci et al. 2023; Santoyo-Garcia et al. 2023). It was noteworthy that the LR6 strain was a genetically engineered variant, specifically designed for T5OAc production via extensive metabolic engineering (Walls et al. 2020). In bioreactor condition, the strain's T5OAc production was limited to 3.7 mg/L and was undetectable in shake-flask culture. This underscored the necessity for multiple rounds of metabolic engineering and optimization to develop highly efficient T5OAc-producing strain. In contrast, *E. coli* was reported to produce up to 1 g/L of Tax, highlighting its potential as a host for synthesizing paclitaxel intermediates (Ajikumar et al. 2010). However, research on T5OAc production in *E. coli* remained scarce, only less than 1 mg/L of T5OAc was detected in *E. coli* MG1655 (DE3) (Edgar et al. 2016). The primary reason for achieving such low titer was attributed to the utilization of a chassis that was not the optimized engineered strain, referred to as strain 7, known for its high production of oxygenated taxanes (Biggs et al. 2016). Additionally, another factor was the absence of fermentation conditions optimization. Based on prior research, *E. coli* MG1655 (DE3) exhibited the highest titers of Tax and oxygenated taxanes (Ajikumar et al. 2010; Biggs et al. 2016), indicating that *E. coli* was suitable as a powerful host for T5OAc production. Furthermore, the ratio of T5OH to the total oxygenated taxanes was 26% in *E. coli* BL21 (DE3) through regulation, a value surpassing the 7% observed in MG1655 (DE3). (Wu et al. 2022; Edgar et al. 2016). In view of the above facts, *E. coli* BL21 (DE3) was strategically selected as the host organism for the biosynthesis of T5OAc.

2. Improvements to Figure 1: To enhance clarity, the font size of the chemical structures in Figure 1 should be increased. All abbreviations used in the manuscript, including those for enzymes and compounds, must be clearly shown in the figure. The caption requires careful revision for accuracy and completeness. Additionally, the full name of *E. coli* should be used initially in the manuscript, followed by its abbreviation in subsequent mentions.

Response: We have meticulously revised the figures, abbreviations, and captions for accuracy and clarity.

3. Verification of Enzyme Expression: The current method measures enzyme expression levels via plasmid and promoter copy numbers. Given that some enzymes originate from plants and may form inclusion bodies, an SDS-PAGE analysis is recommended to confirm expression levels, especially active enzymes.

Response: We have supplemented the SDS-PAGE experiment to verify the expression level of the active enzyme, and the results are presented in the Supplementary Figure 13. A simple analysis was presented in the discussion section.

4. Genetic Integration into *E. coli* Genome: The manuscript notes that an increased number of plasmids impacts *E. coli* growth. The authors should consider integrating these genes directly into the *E. coli* genome to potentially alleviate this issue in later work.

Response: In the Discussion section, we have added that further enhancement of T5OAc production could be achieved by integrating enzymes into the genome to reduce metabolic stress.

Reviewer #2: Paclitaxel is a famous anticancer drug, but its bioproduction mainly relies on the extraction from mature Taxus trees with very-low titer. Scientists have never stop pursuing novel biosynthetic methods to produce paclitaxel by microbial cell factories. However, as the natural biosynthetic pathway of paclitaxel is highly complicated, production of its intermediates has become a compromise solution.

This study proposed a biosynthesis method for the key intermediate taxadien-5α-yl-acetate by *E. coli*. With the systematic metabolic engineering strategies of enhancing enzyme expression, releasing metabolic burden and modulizing and balancing pathway, the authors achieved the reported highest titer of the target compound in engineered *E. coli*.

Some minor points:

1. Line 287-289, if lowering copy numbers can relieve cell metabolic burden, why strain EBTA23 carried low-copy plasmid and EBTA24 carried high-copy plasmid?

Response: In general, high-copy plasmids, compared to low-copy ones, tend to impose greater metabolic stress on strains due to their higher expression levels. In our study, to investigate the impact of a single plasmid system on the production of T5OAc, we chose the low-copy plasmid pACYC Duet-1 and the high-copy plasmid pRSF Duet-1. The results indicated that the strain EBTA23, carrying the low-copy plasmid, had a higher titer than the high-copy strain EBTA24. This can primarily be attributed to metabolic balance, a conclusion also supported by subsequent Multivariate-Modular Metabolic Engineering. Since the production of T5OAc involves multiple enzymes, and the appropriate expression between each enzyme is crucial for the titer, we divide the metabolic pathway into modules and optimize each module in the following study to maximize the production of T5OAc. Compared to EBTA24, EBTA23 showed lower expression levels of all enzymes, which may result in relatively better coordination among them for the titer of T5OAc. Thus, it was logical that EBTA23 demonstrated a higher titer.

2. Figure 1, chemical structure is not clear. It is better to reconstruct this figure. the same goes for graphical abstract.

Response: We have reconstructed Figure 1 and Graphical Abstract.

3. Figure 2 and 3, please add statistical mark in the corresponding figures, despite that some were mentioned in the legends.

Response: Statistical markers have been added at the corresponding figures.

4. Figure 3a, it is better to show a control without metabolic burden caused by plasmids.

Response: Thank you for your suggestion. However, we think that a plasmid-free strain is not necessary in our context. All of our constructed recombinant strains are based on plasmid expression. In our experiments aimed at reducing metabolic stress, we optimized the quantity of plasmids. The results indicated that a reduction in the number of plasmids led to enhanced growth of the strains. These data are already sufficient. However, if a plasmid-free control strain were to be introduced for variable control, it should not be an unmodified *E. coli* BL21 (DE3) but rather a strain that expresses DXS, IDI, TS, GGPPS, T5αOH, TAT at the genomic level, implying that genomic integration would replace plasmid-based expression. This is a subject of our subsequent studies, as mentioned in the Discussion section. These are our reasons, and we sincerely request your understanding.

5. If possible, further polish the language.

Response: We have polished some parts of the article.

Reviewer #3: The authors through the manuscript titled "Enhancing the Biosynthesis of Taxadien‐5α‐yl‐acetate in *Escherichia coli* by Combinatorial Metabolic Engineering Approaches" demonstrated significant improvement in titer for a paclitaxel with the help of modular metabolic engineering approach. While the article is well written and the results are presented in a logical manner, there are a few questions that arise which would help in evaluating the manuscript further:

1. The parameter optimization with the strain emerged from the modular engineering was unsuccessful at the scale-up stage. What was the reason for this? Why is the trade-off between growth and productivity existing in these engineered strains?

Response: There is an optimal combination for multi-parameter optimization. In order to detect the target product as much as possible during the initial fermentation of the strain, we tentatively determined a fermentation parameter after referring to several literatures on the synthesis of paclitaxel intermediates (Chau et al. 2016; Wu et al. 2022; Wang et al. 2022). It is also reasonable that there is no positive effect in the subsequent parameter optimization, because the initial parameters are already suitable for T5OAc fermentation production. Subsequently, we scaled up the culture at the bioreactor level to increase the titer of T5OAc from 5.1mg/L to 10.9mg/L. For trade-off between growth and productivity, we think that higher glycerol may make the strain grow faster, and the pyruvate produced will flow more to the growth pathway. However, since the production of T5OAc also requires pyruvate as a precursor, there will be a trade-off between growth and productivity. This phenomenon is not uncommon. In Liu et al. 's study on the production of adipic acid, the growth of the strain became better and better with the increase of carbon source concentration, but the titer showed a downward trend (Liu et al. 2023).

2. How were the targets for metabolic engineering identified? Please describe the steps taken to arrive at the targets in detail.

Response: Metabolic engineering aims to produce specific target products by altering cell characteristics or constructing new synthetic pathways. As detailed in our manuscript, paclitaxel, a prominent anti-cancer drug molecule with a vast market, currently faces production limitations due to scarce resources. Directed modification of microorganism through metabolic engineering holds the promise for biosynthesis of paclitaxel. However, the complete biosynthetic pathway of paclitaxel remains unresolved. In our research, we focused on T5OAc, an early intermediate in Taxol synthesis, which is understudied and produced in low titer. Our objective is to construct an efficient cell factory for T5OAc biosynthesis, laying a strong foundation for subsequent intermediate synthesis. To enhance T5OAc titer, we employed a series of metabolic engineering strategies. At first, we expressed all pathway enzymes on plasmids, establishing a synthetic pathway in *E. coli* for T5OAc production. Then, we explored the rate-limiting enzymes of *E. coli*'s MEP pathway to provide ample precursors for T5OAc, followed by optimization of the plasmid's production system in terms of quantity and copy number. Metabolic balance is crucial for the biosynthesis of long pathway, so we adopted a multivariate-modular metabolic engineering approach to modularize the synthetic pathway for better regulation of pathway enzymes, and obtained 5.1mg/L T5OAc at shake-flask. Finally, the titer was further increased to 10.9mg/L through the fed-batch fermentation in a 5-L bioreactor. This is the highest reported T5OAc production of *E. coli*.

3. Why was the pathway divided into modules? Can this method be compared to a more generic method where a group of targets are identified by lumping all three modules?

Response: As detailed in the manuscript, during the initial construction of the strain producing T5OAc, we observed an accumulation of the precursor T5OH, which was further addressed by regulating the expression of the acetyltransferase. It was discovered that even minor variations in acetyltransferase expression markedly influenced the titer of T5OAc. Therefore, the potential impact of expression regulation of other enzymes on the titer of T5OAc merits further investigation. Additionally, the biosynthetic pathway for T5OAc involves the overexpression of six enzymes (DXS, IDI, GGPPS, TS, T5αOH, TAT). In long pathway biosynthesis, metabolic imbalance is common, which preclude maximization of the target product titer. Metabolic imbalance refers to the discordance in the expression levels of enzymes within a biosynthetic pathway, leading to excessive accumulation of intermediates or feedback inhibition, thereby hindering the production of the target compound (Biggs et al. 2014). Multivariate-Modular Metabolic Engineering approaches have been repeatedly demonstrated to modularize metabolic pathways and finely regulate them, achieving better control within a limited combinatorial space. Consequently, in light of the aforementioned reasons, we attempted to enhance the titer of T5OAc by modularizing its synthesis pathway using a Multivariate-Modular Metabolic Engineering approach for refined control. Modularization is a common strategy in metabolic engineering. Typically, in metabolic engineering, strategies to enhance the titer of a target product often involve optimizing the rate-limiting enzymes in the biosynthetic pathway. This can include overexpression, screening, fusion expression, self-assembly, or protein engineering modifications. Additionally, blocking competitive pathways to increase the flux through the synthetic pathway is also employed. Modularization is particularly geared towards optimizing longer biosynthetic pathways. The choice of method or combination of methods depends on the limiting factors in the synthesis of the target product. Sometimes, combining modularization with other metabolic engineering strategies can yield unexpected results. The number of modules for modularization needs to be determined based on specific circumstances, with a typical range being 2-4 modules for control. In our research, we optimized the expression, selection, and fine-tuning of rate-limiting enzymes to develop the strain EBTA25, which was regulated by three promoters. Following this, modularization was implemented using strain EBTA25 as the foundational basis. Considering that the three promoters individually regulated the synthesis, oxidation, and acetylation of Tax, we divided the biosynthetic pathway into three modules. Subsequently, we regulated these modules to enhance the titer of T5OAc.

**REFERENCES**

Biggs BW, Paepe BD, Santos CNS, Mey MD, Ajikumar PK (2014) Multivariate modular metabolic engineering for pathway and strain optimization. *Curr Opin Biotechnol 29C* (1): 156-162. Doi: 10.1016/j.copbio.2014.05.005

Chau MD, Walker K, Long R, Croteau R (2004) Regioselectivity of taxoid-*O*-acetyltransferases: heterologous expression and characterization of a new taxadien-5α-ol-*O*-acetyltransferase. *Arch Biochem Biophys 430* (2): 237-246. Doi: 10.1016/j.abb.2004.07.013

Liu J, Gao C, Chen XL, Guo L, Song W, Wu J, Wei WQ, Liu J, Liu LM (2023) Metabolic engineering of *Escherichia coli* for adipic acid production. *J Biotechnol* *39* (6): 2375-2389. Doi: 10.13345/j.cjb.220992

Wang JY, Huang ZY, Wu QY, Pan J, Li CX, Xu JH (2022) Facile biosynthesis of taxadiene by a newly constructed *Escherichia coli* strain fusing enzymes taxadiene synthase and geranylgeranyl pyrophosphate synthase. *Process Biochem* *122*: 129-136. Doi: 10.1016/j.procbio.2022.09.005

Wu QY, Huang ZY, Wang JY, Yu HL, Xu JH (2022) Construction of an *Escherichia coli* cell factory to synthesize taxadien-5 alpha-ol, the key precursor of anti-cancer drug paclitaxel. *Bioresour Bioprocess* *9* (1): 82. Doi: 10.1186/s40643-022-00569-5
